# Targeting Ferroptosis in Neurodegenerative Diseases: Therapeutic Potential and Mechanistic Insights of Traditional Chinese Medicine

**DOI:** 10.3390/biology15191760

**Published:** 2026-10-02

**Authors:** Pan Li, Zi-Meng Qi, Jing-Zhen Wu, Jia Liu, Peng Yu

**Affiliations:** 1 School of Pharmacy, Changchun University of Chinese Medicine, Jilin 130117, China; 25104800147@ccucm.edu.cn (P.L.); 25204897138@ccucm.edu.cn (Z.-M.Q.); 25204897140@ccucm.edu.cn (J.-Z.W.); 2 Scientific Research Department, Changchun University of Chinese Medicine, Jilin 130117, China; 3 Innovation Practice Center, Changchun University of Chinese Medicine, Jilin 130117, China

**Keywords:** ferroptosis, neurodegenerative diseases, traditional Chinese medicine, iron metabolism, lipid peroxidation, glutathione peroxidase 4, Nrf2, acupuncture

## Abstract

Neurodegenerative diseases (NDs), including Alzheimer’s disease, Parkinson’s disease, Huntington’s disease, amyotrophic lateral sclerosis, and multiple sclerosis, are characterized by progressive and irreversible neuronal loss, whereas current treatments remain largely symptomatic. Ferroptosis is an iron-dependent form of regulated cell death whose execution is defined by iron-enabled phospholipid peroxidation and failure of cellular ferroptosis-defense systems. Mitochondrial metabolism can modify ferroptosis susceptibility, but its contribution varies with cell type and initiating stimulus. Converging evidence implicates ferroptosis in multiple NDs, in which affected regions commonly show iron accumulation, glutathione depletion, and increased lipid peroxidation. With their multi-component and multi-target properties, traditional Chinese medicine (TCM) interventions may modulate several nodes in this network. This review summarizes the core execution and defense mechanisms of ferroptosis, evaluates their roles in major NDs, and critically appraises bioactive herbal compounds, compound formulas, acupuncture, electroacupuncture, and moxibustion. The reported interventions mainly engage the Nrf2/GPX4 antioxidant axis, TFR1/FPN1/FTH1/NCOA4-mediated iron handling, ACSL4-dependent lipid peroxidation, and GPX4-independent defense systems. Because most studies are preclinical and many infer ferroptosis from non-specific markers, the evidence is graded here as direct, supportive, indirect, mixed, or complementary. Translation will require standardized formulations, causal ferroptosis validation, pharmacokinetic and brain-exposure data, validated human biomarkers, and rigorously designed clinical trials.

## Simple Summary

Neurodegenerative diseases such as Alzheimer’s disease, Parkinson’s disease, Huntington’s disease, amyotrophic lateral sclerosis, and multiple sclerosis slowly destroy brain cells, and no curative treatment is currently available. Recent studies suggest that these disorders share a common mode of neuronal death, namely ferroptosis, a form of regulated cell death driven by iron overload that causes oxidative damage to lipid molecules in cell membranes. Traditional Chinese medicine (TCM) offers a distinctive approach to counteracting this process. Numerous studies have found that bioactive components of Chinese herbal medicines (such as flavonoids, saponins, and polyphenols), compound formulas, and therapies such as acupuncture, electroacupuncture, and moxibustion can protect neurons by restoring cellular antioxidant defenses, correcting iron homeostasis, and alleviating lipid damage. These interventions have been reported to affect multiple targets, a property that may be relevant to the complex pathology of these disorders. However, current evidence is derived mainly from laboratory and animal studies, and clinical confirmation from patient populations remains lacking. This review summarizes the mechanisms by which ferroptosis drives neurodegenerative diseases and its regulation by TCM, aiming to provide a scientific basis for the development of innovative TCM-based therapies and to highlight the urgency of advancing formulation standardization and high-quality clinical trials.

## 1. Introduction

Neurodegenerative diseases (NDs) are a group of heterogeneous disorders characterized by the progressive and irreversible loss of neuronal structure and function in the brain and spinal cord, including Alzheimer’s disease (AD), Parkinson’s disease (PD), Huntington’s disease (HD), amyotrophic lateral sclerosis (ALS), and multiple sclerosis (MS) [1]. With accelerating global population aging, the burden of NDs continues to grow. Taking Alzheimer’s disease as an example, the GBD 2019 model projects that the global prevalence of all-cause dementia will reach 152.8 million (95% UI 130.8–175.9 million) by 2050 [2], while the WHO estimate based on demographic projections is 139 million for the same period [3]. As the most common subtype (accounting for 60% to 70% of cases), AD is expected to affect approximately 90 to 100 million people [4], imposing an enormous socioeconomic burden. Despite decades of research, current therapeutic strategies remain largely limited to symptomatic relief [5] and cannot halt or reverse the underlying neuronal loss, highlighting the urgent need to develop disease-modifying therapies that target the core mechanisms of neurodegeneration.

Ferroptosis, an iron-dependent form of regulated cell death first described by Dixon et al. in 2012 [6], has emerged as a candidate executor of neuronal injury in multiple NDs [7]. It is morphologically, biochemically, and genetically distinct from apoptosis and necroptosis. Its execution is organized around three tightly coupled modules: expansion of redox-active iron, enzymatic and non-enzymatic peroxidation of polyunsaturated phospholipids, and failure of GPX4-dependent or GPX4-independent antioxidant defenses [8,9,10,11,12,13,14]. Mitochondria can amplify or restrain these processes through metabolism, iron handling, and radical-trapping systems in a context-dependent manner [15,16]. Brain tissues from patients and disease models frequently show combinations of iron accumulation, glutathione depletion, reduced antioxidant capacity, and lipid-peroxidation products. These convergent features support ferroptosis as a shared, potentially druggable mechanism whose contribution varies across cell types, disease stages, and injury contexts.

TCM, characterized by multiple components, multiple targets, and multiple pathways, has been investigated as a multi-target approach for regulating ferroptosis. Its material basis encompasses bioactive components of individual herbs (flavonoids, glycosides, phenolics, polysaccharides, terpenoids, and lignans), the multi-component pharmacological networks of compound formulas, and physical stimulation therapies such as electroacupuncture, acupuncture, and moxibustion. Mechanistically, these interventions converge on restoring the Nrf2/GPX4 antioxidant axis, correcting iron homeostasis (TFR1/FPN1/FTH1/NCOA4), inhibiting ACSL4-mediated lipid peroxidation, and modulating transcription factors such as Nrf2 and p53, thereby rebalancing pro-death and anti-death signals within neurons. Although numerous preclinical studies have reported the anti-ferroptotic and neuroprotective effects of single compounds, compound formulas, and TCM-derived therapies, a comprehensive review that systematically integrates the molecular mechanisms of ferroptosis with the multidimensional regulatory capacity of TCM remains lacking.

This review first summarizes the core mechanisms of ferroptosis and its key roles in the pathological progression of major NDs. It then reviews the anti-ferroptotic effects of the bioactive components of Chinese herbal medicines, compound formulas, and characteristic TCM therapies in NDs, together with the underlying molecular networks. Our aim is to provide a theoretical foundation and new perspectives for the development of TCM-based therapeutic strategies for neurodegenerative diseases.

### Literature Identification and Scope

This article is a narrative review. PubMed, Web of Science, and China National Knowledge Infrastructure (CNKI) were searched, with the final literature update covering records available through August 2026. The core search combined “ferroptosis” with disease terms (“neurodegenerative disease”, “Alzheimer’s disease”, “Parkinson’s disease”, “Huntington’s disease”, “amyotrophic lateral sclerosis”, or “multiple sclerosis”) and intervention terms (“traditional Chinese medicine”, “natural compound”, “acupuncture”, “electroacupuncture”, or “moxibustion”); equivalent Chinese terms were used in CNKI. Disease- or intervention-specific searches combined the relevant term with “ferroptosis”. Searches were supplemented by reference-list screening and the targeted retrieval of mechanistically relevant studies. English- and Chinese-language publications were considered. Selection proceeded in two stages: records were first assessed for relevance to ferroptosis, the five core neurodegenerative diseases, or TCM interventions, and eligible original reports were then assigned to Table 1, Table 2, Table 3, Table 4, Table 5, Table 6, Table 7, Table 8, Table 9, Table 10, Table 11 and Table 12 when they reported at least one ferroptosis-related readout together with a functional or survival outcome. Mechanistic and clinical studies of ferroptosis in the five diseases were prioritized, while representative reviews were used for background context. Editorials, conference abstracts without sufficient data, and reports not addressing ferroptosis were not used as evidence. The final bibliography contains 212 cited publications.

Because markers such as ROS, MDA, GSH, GPX4, SLC7A11, or Nrf2 are not individually specific to ferroptosis and may also reflect general oxidative stress or other forms of regulated cell death, we applied a five-level evidence framework to all 116 table entries. Direct evidence required a lipid-peroxidation readout (for example, C11-BODIPY, 4-HNE, or MDA) together with selective pharmacological rescue or a causal, pathway-specific genetic perturbation that reversed or abolished the intervention effect. Supportive evidence included ferroptosis-inducer or inhibitor benchmarking, or mechanistic perturbation that validated the model but did not causally test the intervention. Indirect evidence comprised changes in ferroptosis-associated markers without selective rescue or causal genetic validation. Mixed evidence denotes table rows that cite studies spanning more than one evidence level or scope, whereas complementary evidence denotes non-core diseases, synthetic TCM-inspired molecules, or studies whose primary endpoint was another cell-death pathway. These definitions are used consistently in Table 1, Table 2, Table 3, Table 4, Table 5, Table 6, Table 7, Table 8, Table 9, Table 10, Table 11 and Table 12 to distinguish causal mechanistic evidence from associative ferroptosis-related findings and from evidence outside the core scope.

## 2. Core Mechanisms of Ferroptosis

**Figure 1:**
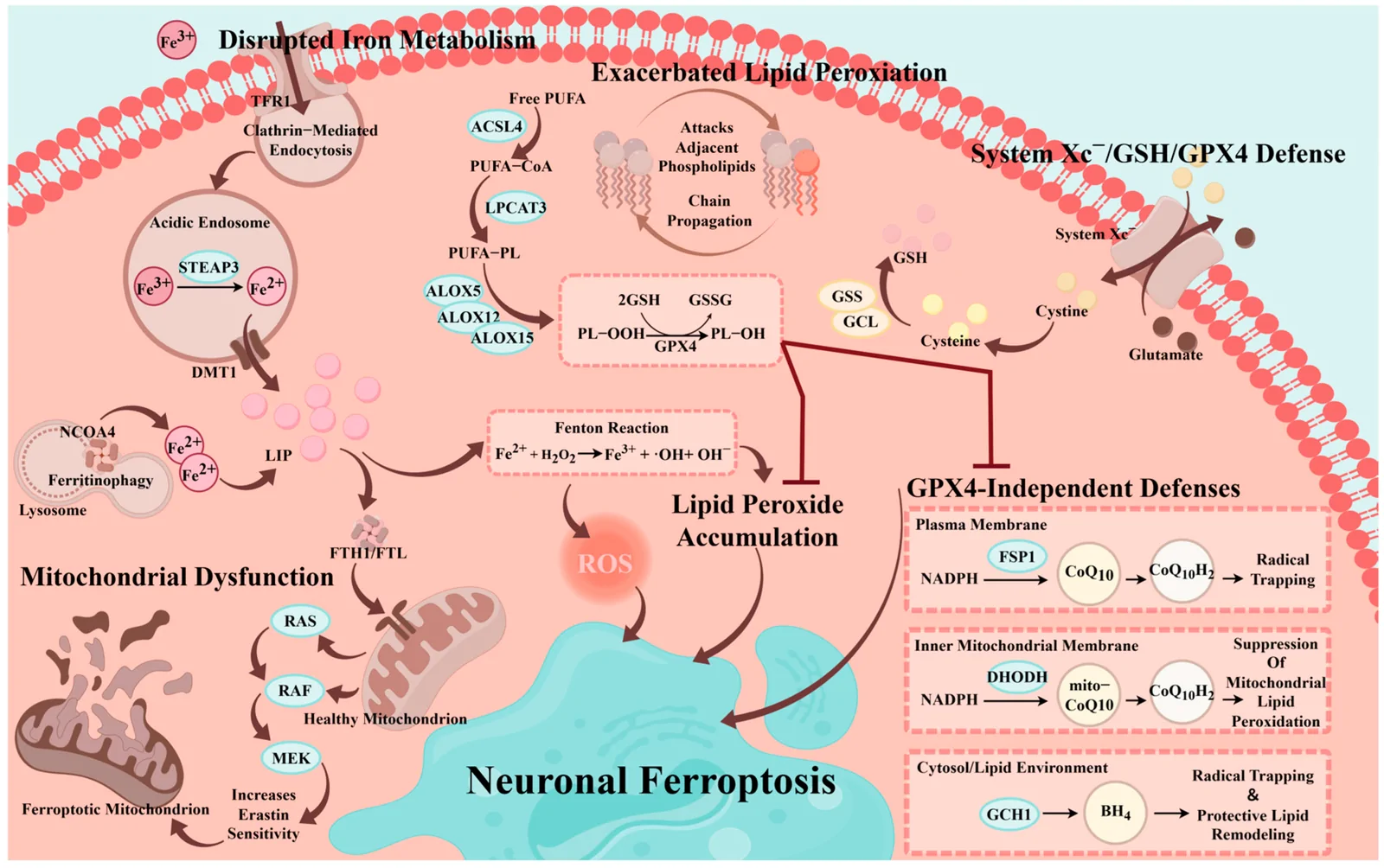
Mechanistic framework of ferroptosis signaling and defense. (1) Iron handling: TFR1-mediated endocytosis, STEAP3, DMT1, ferritin, FPN1, and NCOA4-dependent ferritinophagy regulate the labile iron pool; Fe^2+^ drives Fenton chemistry and promotes radical formation. (2) Phospholipid peroxidation: ACSL4 and LPCAT3 incorporate polyunsaturated fatty acids into membrane phospholipids, which undergo enzymatic and non-enzymatic peroxidation and chain propagation. (3) GPX4-dependent defense: cystine import through System Xc^−^ supports GSH synthesis, enabling GPX4 to reduce phospholipid hydroperoxides (PL-OOH) to phospholipid alcohols (PL-OH). (4) GPX4-independent defense: FSP1/CoQ10, DHODH/mitochondrial CoQ10, and GCH1/BH4 provide parallel radical-trapping systems. (5) Mitochondrial contribution: the diagram depicts mitochondrial dysfunction and the RAS–RAF–MEK–VDAC route; these features modulate ferroptosis susceptibility in a context-dependent manner. Imbalance between phospholipid oxidation and these defense systems culminates in neuronal ferroptosis. Legend: → (Solid arrow): Activation/Promotion. Indicates that the upstream molecule promotes or activates the function of the downstream molecule. ├ (T-bar): Inhibition. Indicates that the upstream molecule inhibits or negatively regulates the downstream molecule.

Ferroptosis is an iron-dependent form of regulated cell death first defined by Dixon et al. in 2012 [6]. Its mechanistic framework comprises the iron-dependent generation and propagation of phospholipid peroxides, the GPX4/GSH system and parallel radical-trapping defense networks that restrain this process, and context-dependent modifiers—including mitochondrial metabolism and genetic regulation—that alter cellular susceptibility (Figure 1).

### 2.1. Disrupted Iron Metabolism

Iron is an essential element for life, but the disruption of intracellular iron homeostasis is a critical prerequisite for the initiation of ferroptosis [17,18]. Extracellular ferric iron (Fe^3+^) binds to transferrin (Tf) and is recognized by transferrin receptor 1 (TFR1) on the cell membrane, entering the cell through endocytosis. In the acidic environment of endosomes, Fe^3+^ is reduced to ferrous iron (Fe^2+^) by STEAP3, and Fe^2+^ is then transported into the cytoplasm by divalent metal transporter 1 (DMT1), forming the labile iron pool (LIP) [19,20]. Fe^2+^ in the LIP is highly unstable and can react with H_2_O_2_ through the Fenton reaction to generate highly reactive hydroxyl radicals (·OH), which in turn trigger membrane lipid peroxidation, disrupt cell membrane structure, and ultimately induce ferroptosis [21,22]. Intracellular iron levels are precisely regulated by ferritin storage and export through ferroportin 1 (FPN1). Ferritin, composed of heavy (FTH) and light (FTL) chains, sequesters Fe^2+^ and prevents it from entering the LIP, whereas FPN1 is currently the only known transporter capable of exporting intracellular iron [23]. A further layer of control comes from nuclear receptor coactivator 4 (NCOA4), which mediates ferritinophagy, the selective degradation of ferritin that releases Fe^2+^ into the LIP and thereby increases susceptibility to ferroptosis [24,25,26]. Therefore, the regulation of LIP content by iron metabolism is a critical determinant of whether ferroptosis occurs, and abnormal expression of iron transport and storage proteins such as TFR1, DMT1, ferritin, and FPN1 is closely associated with ferroptosis [27].

### 2.2. Exacerbated Lipid Peroxidation

Lipid peroxidation is the core execution step of ferroptosis, and its substrates are mainly membrane phospholipids containing polyunsaturated fatty acids (PUFA-PLs) [28]. Free PUFAs alone are insufficient to trigger ferroptosis and must first be incorporated into cell membrane phospholipids to become peroxidation substrates. This process is completed cooperatively by two key enzymes: acyl-CoA synthetase long-chain family member 4 (ACSL4) converts free PUFAs into PUFA-CoA, and lysophosphatidylcholine acyltransferase 3 (LPCAT3) then esterifies PUFA-CoA into membrane phospholipids to form PUFA-PLs [10,29]. ACSL4 is regarded as the gatekeeper enzyme of ferroptosis, and its expression level and activity directly determine cellular sensitivity to ferroptosis. High ACSL4 expression enriches oxidizable PUFAs in the cell membrane and places the cell in a pre-activated state [9,30]. The peroxidation of PUFA-PLs occurs through enzymatic and non-enzymatic routes. The enzymatic route is mainly mediated by lipoxygenases (LOXs, including 5-LOX, 12-LOX, and 15-LOX), iron-containing enzymes that catalyze PUFA oxidation, whereas the non-enzymatic route is driven by the iron-dependent Fenton reaction, which generates large amounts of ROS and initiates PUFA-PL peroxidation [31]. Once generated, lipid peroxides interact with Fe^2+^ to produce peroxyl radicals that further attack adjacent PUFA-PLs, forming a chain propagation that ultimately leads to excessive accumulation of lipid peroxides, disrupts the fluidity and integrity of the cell membrane, and triggers ferroptosis [32]. Therefore, the expression levels of ACSL4 and LPCAT3 are key targets for regulating lipid peroxidation and ferroptosis, and inhibiting ACSL4 can effectively block the onset of ferroptosis [9,33].

### 2.3. Impairment of the GPX4/GSH Antioxidant Defense System

The GPX4/GSH antioxidant defense system is the central cellular line of defense against ferroptosis [11,34]. Glutathione (GSH) is a tripeptide composed of glutamate, cysteine, and glycine, and it is one of the most important antioxidants in mammalian cells [35]. GSH synthesis is highly dependent on the availability of cysteine, which is derived mainly from the uptake of extracellular cystine. This uptake is mediated by the cystine/glutamate antiporter System Xc^−^, which is composed of the light-chain subunit SLC7A11 and the heavy-chain subunit SLC3A2 and transports cystine into the cell while exporting glutamate at a 1:1 ratio [36]. After entering the cell, cystine is rapidly reduced to cysteine, which serves as the rate-limiting substrate for GSH synthesis and is converted to GSH through catalysis by glutamate-cysteine ligase and glutathione synthetase [37]. GPX4 is a selenium-dependent glutathione peroxidase and is currently the only known enzyme capable of directly catalyzing the reduction of membrane-bound phospholipid hydroperoxides (PL-OOH). Using GSH as a cofactor, it reduces toxic lipid hydroperoxides to non-toxic lipid alcohols (PL-OH), thereby terminating the lipid peroxidation chain reaction and effectively suppressing ferroptosis [11,38]. When System Xc^−^ function is inhibited (for example, by erastin) or GSH synthesis is blocked, GPX4 activity is lost. Inactive GPX4 cannot clear the lipid peroxides accumulating within the cell, so lipid peroxides continue to accumulate until they reach a toxic threshold and ultimately trigger the ferroptotic program [39]. Therefore, the System Xc^−^/GSH/GPX4 axis constitutes a key target for defense against ferroptosis, and failure of any of its components removes the final restraint on ferroptosis.

### 2.4. Context-Dependent Mitochondrial Modulation of Ferroptosis

Mitochondria can promote or restrain ferroptosis depending on the initiating stimulus, metabolic state, and cell type [15]. Mitochondrial shrinkage, increased membrane density, and reduced cristae are characteristic ultrastructural features, but morphology alone is not diagnostic and must be interpreted together with lipid-peroxidation measurements and pharmacological or genetic evidence [16]. In some settings, erastin-sensitive voltage-dependent anion channels (VDACs) alter mitochondrial ion and metabolite flux, whereas tricarboxylic-acid-cycle activity and electron transport can enhance lipid-peroxide production during cysteine deprivation [15,40]. The mitochondrial contribution is context-dependent, and mitochondria are dispensable when GPX4 is inhibited directly [15]. Mitochondrial phospholipid peroxidation and iron accumulation can nevertheless amplify respiratory dysfunction and reactive-oxygen-species generation. Conversely, CISD1 and mitochondrial ferritin (FtMt) restrict mitochondrial iron availability, and the mitochondrial CoQ10-reducing enzyme DHODH provides a parallel antioxidant defense [13,38,41]. Mito-TEMPO also blocks doxorubicin-induced cardiac ferroptosis in mice, supporting a functional mitochondrial contribution in that specific context [42]. Accordingly, the RAS–RAF–MEK–VDAC relationship represents a context-dependent route that modulates ferroptosis susceptibility.

### 2.5. GPX4-Independent Ferroptosis Defense Systems

Beyond the System Xc^−^/GSH/GPX4 axis, cells possess GPX4-independent mechanisms that suppress ferroptosis in parallel [12,13,14]. Ferroptosis suppressor protein 1 (FSP1), formerly known as AIFM2, is recruited to the plasma membrane and uses NAD(P)H to reduce coenzyme Q10 (CoQ10) to ubiquinol, a lipophilic radical-trapping antioxidant that interrupts lipid-peroxidation propagation independently of GPX4 [12]. Within mitochondria, dihydroorotate dehydrogenase (DHODH), an inner-membrane enzyme in pyrimidine biosynthesis, reduces mitochondrial CoQ10 and thereby complements GPX4 in restraining mitochondrial lipid peroxidation [13]. A third parallel system is the GCH1/BH4 axis, in which GTP cyclohydrolase 1 (GCH1) drives the synthesis of tetrahydrobiopterin (BH4), which acts as an endogenous radical-trapping antioxidant and remodels the lipid environment [14]. Among the TCM interventions cataloged here, wolfberry glycopeptide has been reported to protect dopaminergic neurons independently of GPX4, and schisandrin B has been associated with FSP1-dependent defense. Direct engagement of DHODH or GCH1/BH4 remains an open mechanistic question. Nrf2 and its downstream target HO-1 exert context-dependent effects: under conditions of iron overload, excessive HO-1 activity can release iron from heme and promote ferroptosis [43].

### 2.6. Genetic Regulation of Ferroptosis

At the genetic level, ferroptosis is governed by a coordinated network of pro- and anti-ferroptotic genes. Anti-ferroptotic genes include GPX4, SLC7A11, FSP1 (AIFM2), NFE2L2 (encoding Nrf2) and its downstream targets (HMOX1, NQO1, FTH1, and FTL), and GCH1, whereas pro-ferroptotic genes include ACSL4, LPCAT3, ALOX family members, TFRC (encoding TFR1), and NCOA4, which mediates ferritinophagy [9,10,12,13,14,26]. Transcriptional control is centered on Nrf2, which upregulates antioxidant and iron-storage genes; on p53, which represses SLC7A11 transcription and thereby promotes ferroptosis; and on stress-responsive factors such as ATF4 and HIF-1α, which modulate amino-acid metabolism and iron handling [11,43]. Post-transcriptionally, the iron-regulatory proteins IRP1 and IRP2 control TFRC and FTH1/FTL mRNAs according to intracellular iron status [17,18]. Epigenetic and non-coding-RNA mechanisms add a further regulatory layer: histone methylation by G9a determines neuronal vulnerability to ferroptosis [44], and miRNAs, lncRNAs, and circRNAs targeting GPX4, SLC7A11, or ACSL4 transcripts have been implicated in neurodegenerative disease models [45]. This genetic framework explains why dysregulated expression of these genes modifies neuronal susceptibility to ferroptosis and provides the molecular basis for the TCM interventions discussed in Section 4.

## 3. Ferroptosis and Neurodegenerative Diseases

**Figure 2:**
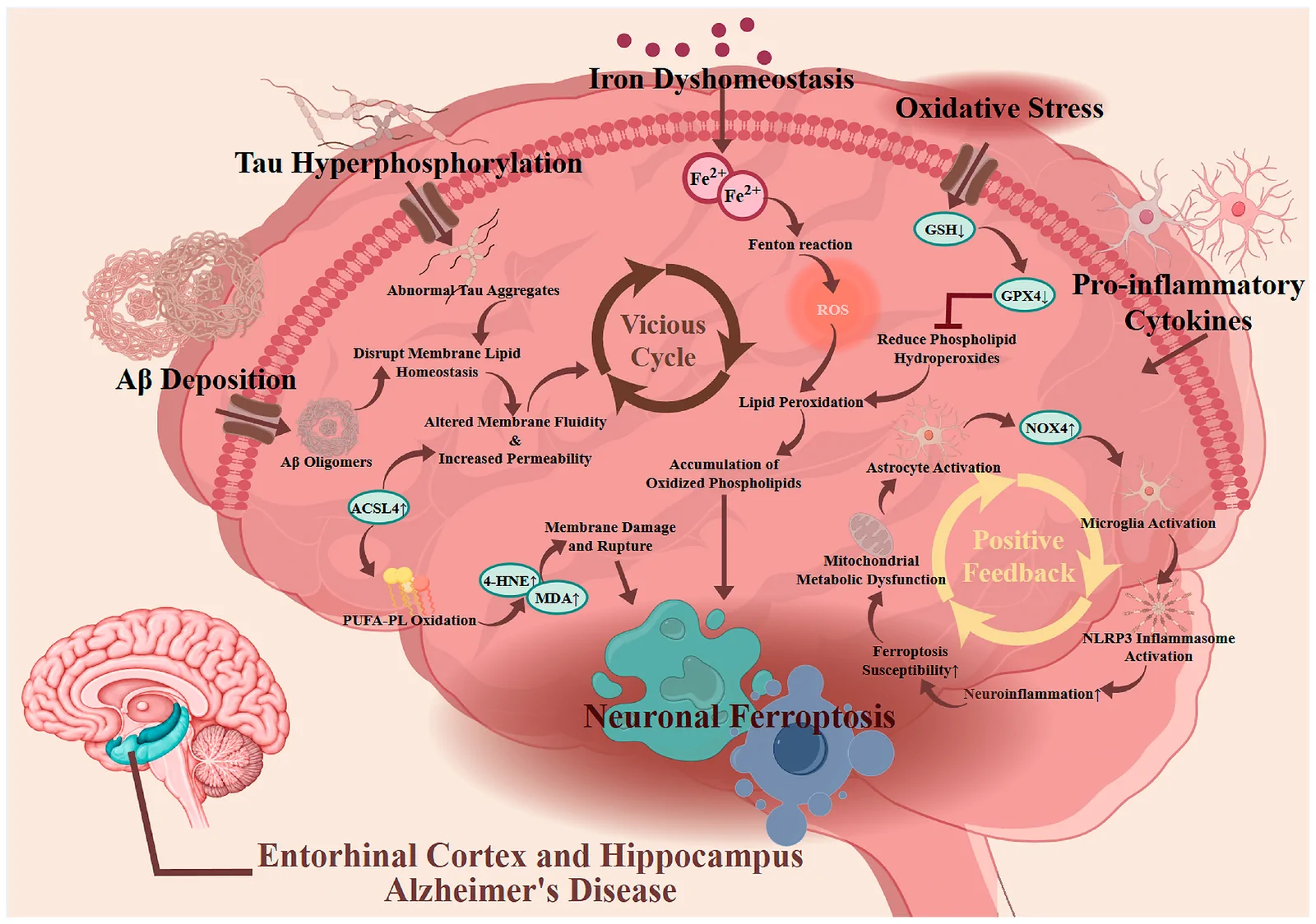
Ferroptosis network in Alzheimer’s disease. Aβ and tau pathology, iron dyshomeostasis, impaired Nrf2–GSH–GPX4 defense, ACSL4-dependent phospholipid peroxidation, glial inflammation, and context-dependent mitochondrial dysfunction form reinforcing loops that promote neuronal ferroptosis and cognitive decline. Legend: → (Solid arrow): Activation/Promotion. Indicates that the upstream molecule promotes or activates the function of the downstream molecule. ├ (T-bar): Inhibition. Indicates that the upstream molecule inhibits or negatively regulates the downstream molecule.

**Figure 3:**
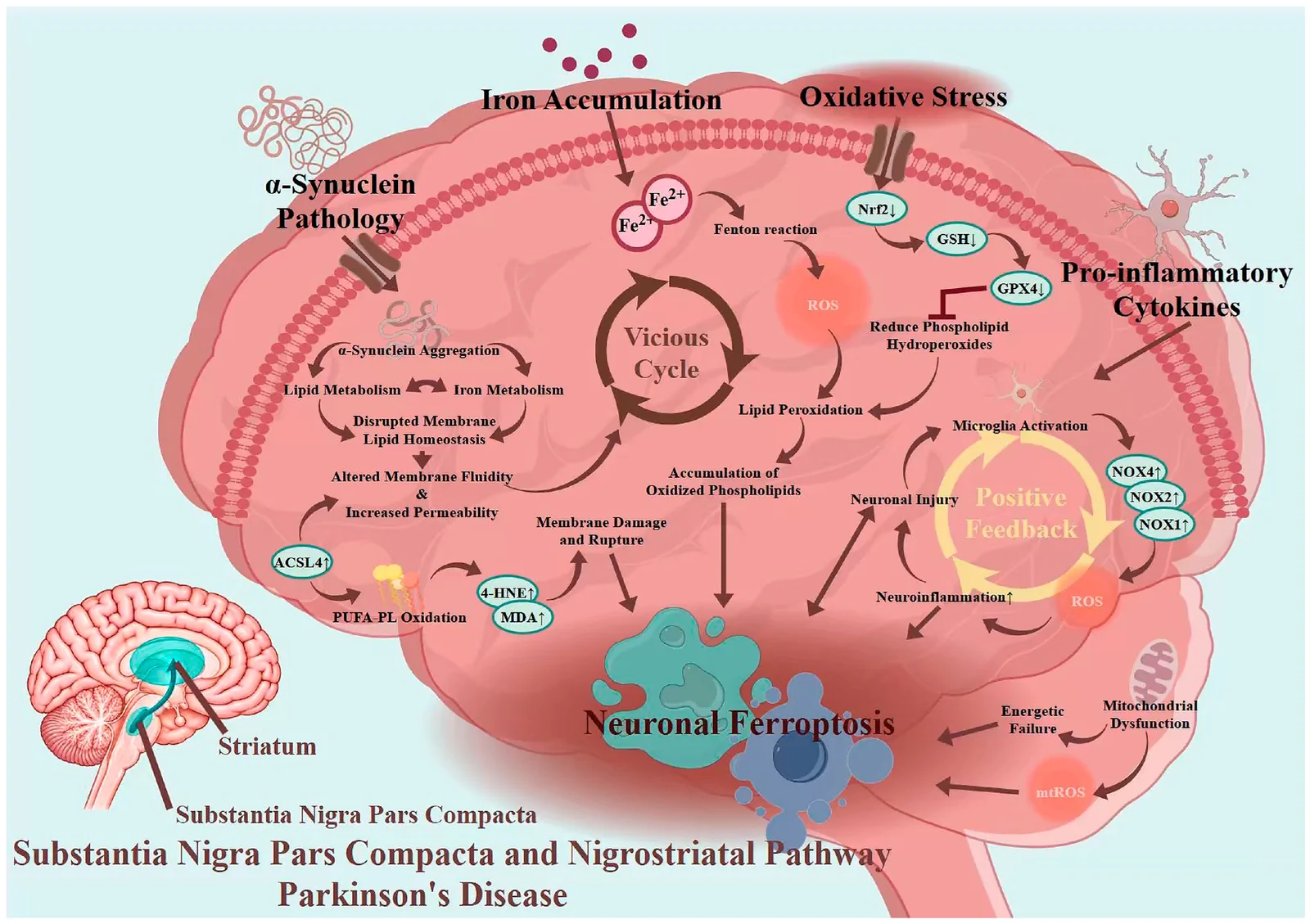
Ferroptosis network in Parkinson’s disease. α-Synuclein accumulation, iron overload, ACSL4-dependent phospholipid oxidation, impaired Nrf2–GSH–GPX4 defense, NOX-driven oxidative stress, glial inflammation, and context-dependent mitochondrial dysfunction converge on dopaminergic-neuron ferroptosis. Legend: → (Solid arrow): Activation/Promotion. Indicates that the upstream molecule promotes or activates the function of the downstream molecule. ├ (T-bar): Inhibition. Indicates that the upstream molecule inhibits or negatively regulates the downstream molecule.

**Figure 4:**
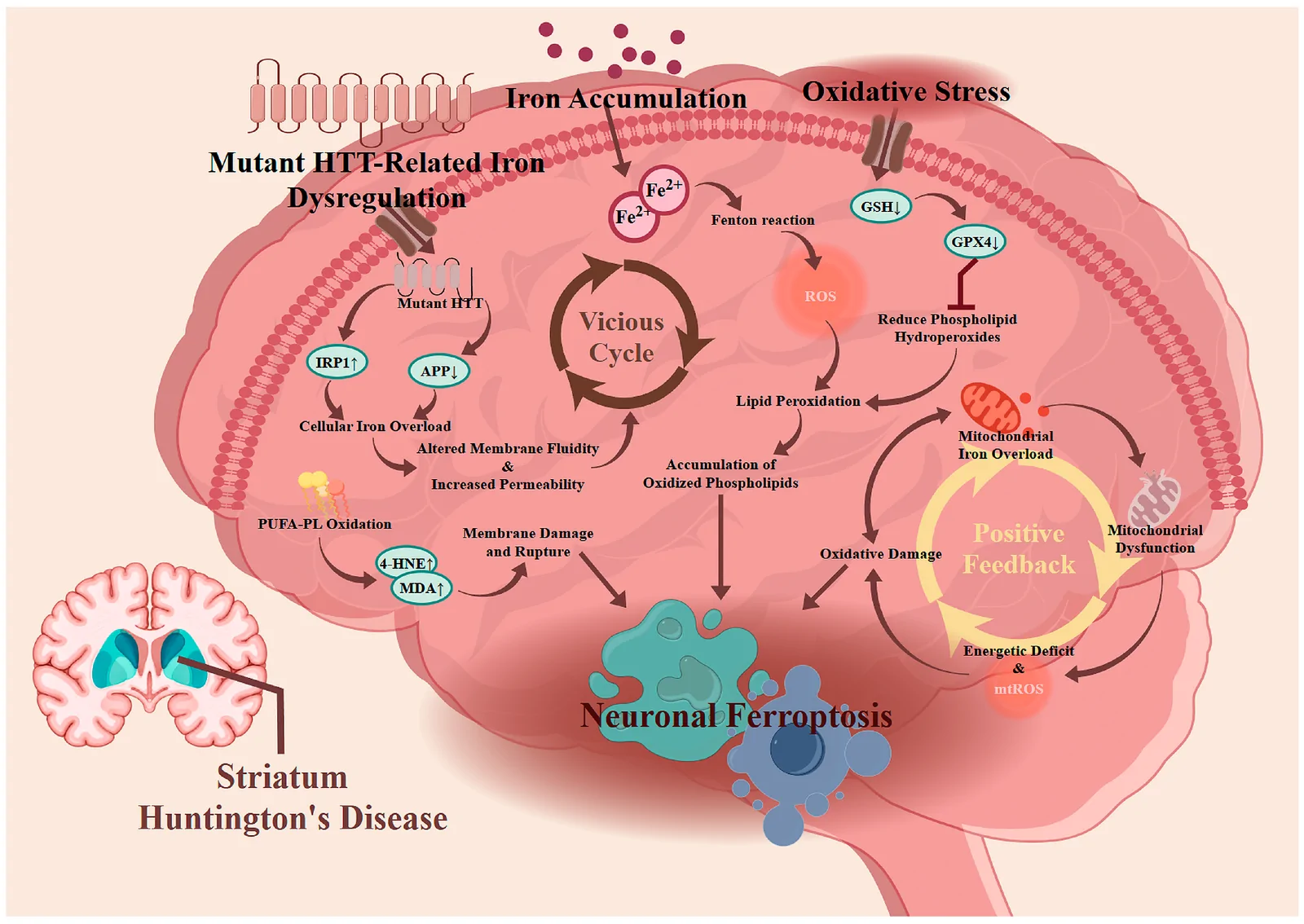
Ferroptosis network in Huntington’s disease. Mutant huntingtin disrupts IRP1- and APP-associated iron handling, promoting cellular and mitochondrial iron accumulation. Fenton chemistry, impaired GSH–GPX4 defense, ACSL4-dependent phospholipid peroxidation, and context-dependent mitochondrial dysfunction then reinforce striatal and cortical neuronal injury. Legend: → (Solid arrow): Activation/Promotion. Indicates that the upstream molecule promotes or activates the function of the downstream molecule. ├ (T-bar): Inhibition. Indicates that the upstream molecule inhibits or negatively regulates the downstream molecule.

**Figure 5:**
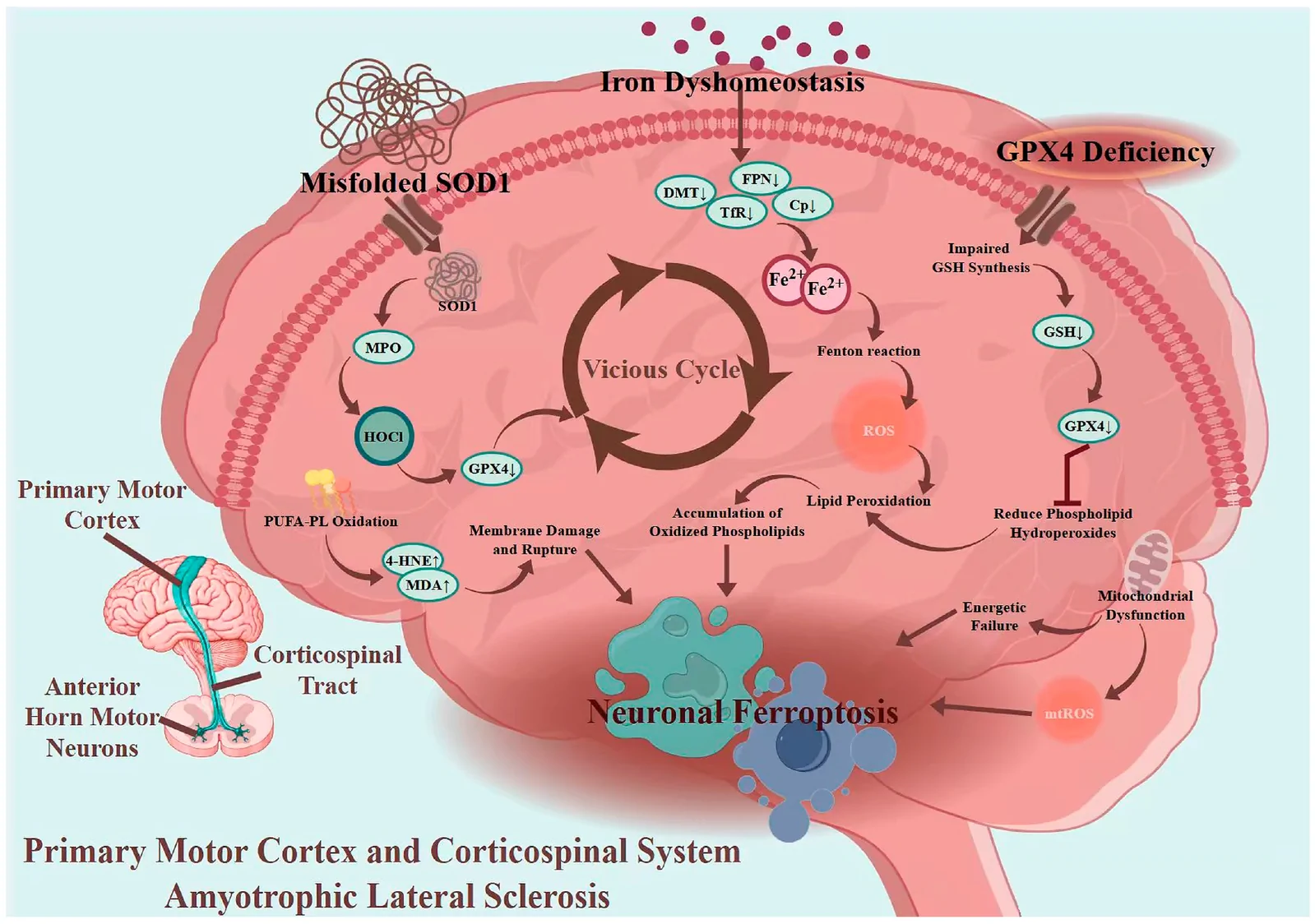
Ferroptosis network in amyotrophic lateral sclerosis. Misfolded SOD1, MPO-derived HOCl, iron dyshomeostasis, reduced GSH–GPX4 activity, phospholipid peroxidation, and context-dependent mitochondrial dysfunction reinforce motor-neuron injury and ferroptotic death. Legend: → (Solid arrow): Activation/Promotion. Indicates that the upstream molecule promotes or activates the function of the downstream molecule. ├ (T-bar): Inhibition. Indicates that the upstream molecule inhibits or negatively regulates the downstream molecule.

**Figure 6:**
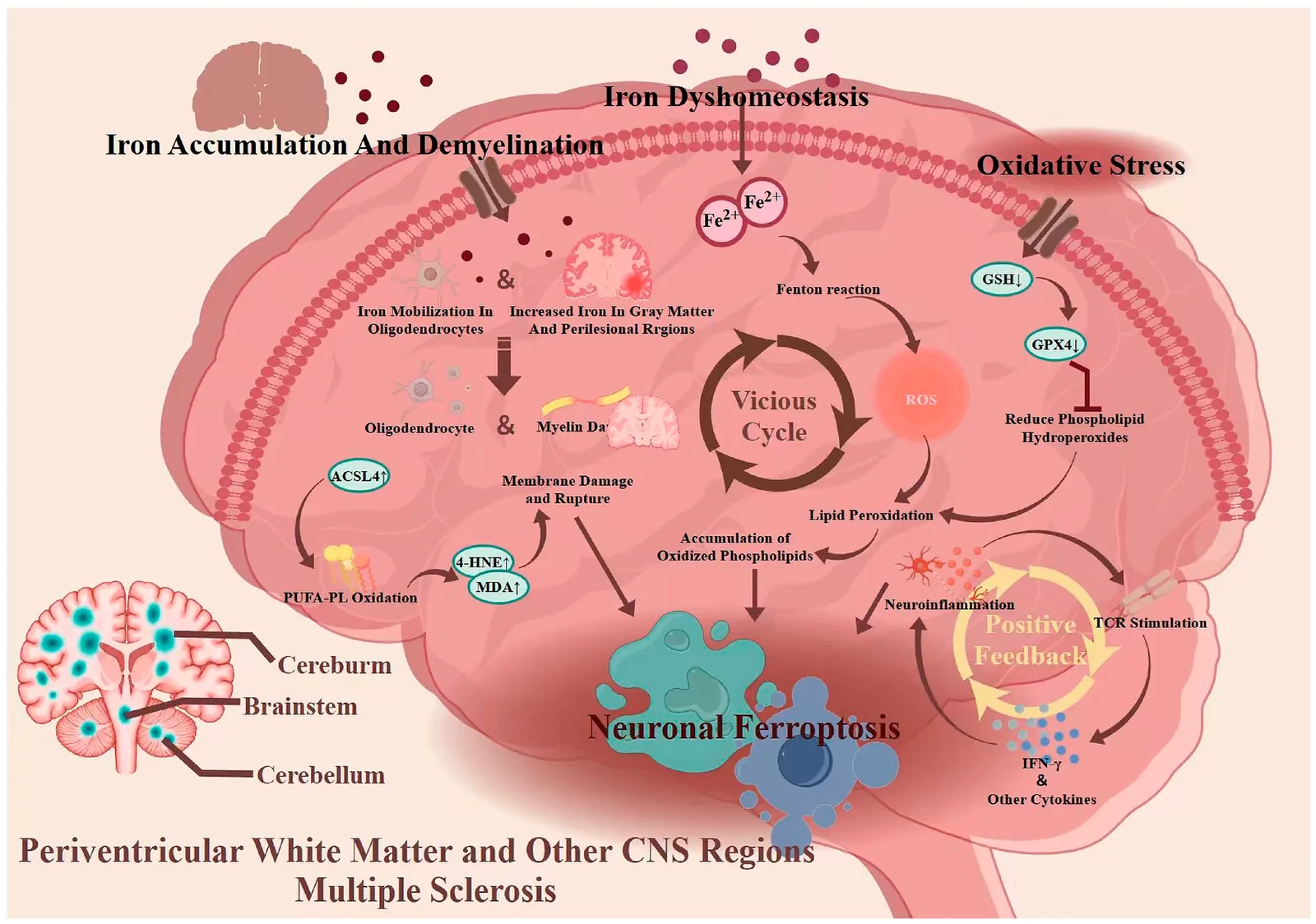
Ferroptosis network in multiple sclerosis. Oligodendroglial iron dysregulation, impaired GSH–GPX4 defense, ACSL4-mediated phospholipid peroxidation, and immune-cell feedback promote ferroptotic injury, demyelination, and neurodegeneration. Legend: → (Solid arrow): Activation/Promotion. Indicates that the upstream molecule promotes or activates the function of the downstream molecule. ├ (T-bar): Inhibition. Indicates that the upstream molecule inhibits or negatively regulates the downstream molecule.

Neurodegenerative diseases (NDs) are a class of disorders characterized by the progressive loss of neuronal structure and function in the brain and spinal cord, mainly including Alzheimer’s disease (AD), Parkinson’s disease (PD), Huntington’s disease (HD), amyotrophic lateral sclerosis (ALS), and multiple sclerosis (MS) [46]. A large body of evidence indicates that ferroptosis plays a key role in the pathological progression of these diseases. Patients commonly exhibit elevated iron levels, reduced glutathione levels, and increased lipid peroxidation in the brain, suggesting that ferroptosis is a shared driver of neuronal death across multiple neurodegenerative diseases [47]. The upstream triggers and dominant mechanisms of ferroptosis differ among diseases, as illustrated by the vicious cycle of Aβ/Tau pathology and iron overload in AD [43] and by the interaction between α-synuclein and iron that aggravates pathological progression in PD [48]. Despite this diversity, disrupted iron metabolism, surging lipid peroxidation, and impaired antioxidant defense systems constitute a common pathway by which ferroptosis drives neurodegeneration. Therefore, targeting ferroptosis is being investigated as a therapeutic strategy for these neurodegenerative diseases [7,49]. On this basis, Figure 2, Figure 3, Figure 4, Figure 5 and Figure 6 depict, for each disease, the shared ferroptosis core together with its disease-specific triggers and crosstalk, so that readers can navigate directly to the disease of interest.

### 3.1. Ferroptosis and Alzheimer’s Disease (AD)

Alzheimer’s disease (AD) is a chronic and progressive neurodegenerative disease characterized by gradually worsening decline in learning and memory and by cognitive dysfunction [50]. According to statistics, there were approximately 26.6 million AD patients worldwide in 2006, and this number is projected to exceed 100 million by 2050 [51]; when all-cause dementia is included, the projection rises to more than 150 million [2]. AD is classified into early-onset and late-onset forms according to age at onset. Early-onset AD usually develops before the age of 65 and is closely associated with genetic mutations in presenilin or amyloid precursor protein (APP). In contrast, late-onset AD mainly affects elderly individuals aged 65 years and older and accounts for approximately 95% of all AD cases [50]. AD is the most common neurodegenerative disease, and its core pathological features are the deposition of β-amyloid (Aβ) plaques and neurofibrillary tangles (NFTs) formed by hyperphosphorylated Tau protein [52]. Ferroptosis is deeply involved in the pathological progression of AD through multiple mechanisms. At the upstream level, disruption of iron homeostasis serves as the initiating event. Patients with AD show marked abnormal iron accumulation in brain regions such as the hippocampus, cortex, and basal ganglia, and clinical MRI studies have confirmed that hippocampal iron content is positively correlated with the degree of tissue damage [53,54]. Iron overload generates large amounts of ROS through the Fenton reaction, directly promotes Aβ deposition, and upregulates β-secretase by inhibiting furin activity, thereby accelerating Aβ production. Iron also promotes Tau hyperphosphorylation through the GSK-3β pathway, forming a vicious cycle between Aβ/Tau pathology and ferroptosis [55,56,57]. Downstream of iron overload, lipid peroxidation constitutes the core execution step of ferroptosis. The lipid peroxidation markers 4-HNE and MDA are significantly elevated in the AD brain, and ACSL4-mediated peroxidation of PUFA-PLs aggravates membrane damage [16,58]. In parallel, dysfunction of the GSH/GPX4 axis acts as a key switch that unleashes ferroptosis. GSH levels are significantly reduced in the hippocampus and cortex of AD patients, GPX4 downregulation is negatively correlated with disease severity, and mice with conditional GPX4 knockout exhibit hippocampus-dependent memory impairment and elevated levels of typical ferroptosis markers [59]. Beyond these core alterations, upregulated NOX4 expression in astrocytes enhances susceptibility to ferroptosis by inducing mitochondrial metabolic dysfunction, whereas NLRP3 inflammasome activation in microglia further amplifies the positive feedback loop between neuroinflammation and ferroptosis [60,61]. Figure 2 shows a schematic illustration of neuronal ferroptosis mechanisms and the vicious cycle in AD progression.

### 3.2. Ferroptosis and Parkinson’s Disease (PD)

PD is the second most common neurodegenerative disease and is characterized by the degeneration of dopaminergic (DA) neurons in the substantia nigra pars compacta and the formation of Lewy bodies [62,63]. The prevalence of PD increases with age; it is approximately 1% in individuals over 65 years of age, peaks around the age of 60, and is about 1.4 times higher in men than in women [64,65]. The core mechanisms of ferroptosis in PD can be summarized as follows. Abnormal brain iron accumulation is a prominent pathological feature of PD. Significant iron deposition is found in the substantia nigra, globus pallidus, and caudate nucleus of PD patients, and iron drives oxidative stress through the Fenton reaction, selectively damaging dopaminergic neurons [62]. Equally important, impairment of the GPX4/GSH system is a key driver of ferroptosis in PD. Ferroptosis was first implicated in the death of dopaminergic neurons in 2016, and specific deletion of GPX4 from dopaminergic neurons leads to PD-like anxiety symptoms and behavioral deficits [59,66]. Neurotoxins commonly used in PD models, such as MPTP and rotenone, can induce ferroptosis, manifested as iron accumulation, reduced GSH levels, and increased lipid peroxides, all of which conform to the biochemical characteristics of ferroptosis [67,68]. With respect to lipid peroxidation, the interaction of α-synuclein with iron and lipid metabolism further aggravates the ferroptotic process [48]. Moreover, NOX family NADPH oxidases (including NOX1, NOX2, and NOX4) exacerbate PD pathology by promoting neuronal ferroptosis and neuroinflammation, and microglial CR3 promotes ferroptosis through NOX2-mediated iron deposition [69]. Taken together, ferroptosis in PD is regulated by multiple interrelated pathways, including the GPX4/GSH system, iron and lipid metabolism, Nrf2 signaling, and mitochondrial function (Figure 3).

### 3.3. Ferroptosis and Huntington’s Disease (HD)

HD is an autosomal dominant inherited neurodegenerative disease caused by CAG trinucleotide repeat expansion in the HTT gene and is characterized by neurodegeneration of the striatum and cortex [70,71]. HD is a relatively rare neurodegenerative disease, with a prevalence of approximately 5.7 per 100,000 in European and American populations and only about 0.4 per 100,000 in Asian populations [72]. HD usually develops in adulthood (approximately 30 to 50 years of age) and is characterized by degeneration of striatal and cortical neurons, with pathology that is highly correlated with the genetic background. Several lines of evidence delineate the role of ferroptosis in HD, among which iron accumulation serves as a key trigger. MRI studies have shown significantly elevated iron levels in the caudate nucleus, putamen, and globus pallidus of HD patients. Iron and ferritin levels are increased in the basal ganglia and striatum of HD patients, and elevated iron levels have also been observed in the cortex and striatum of R6/2 HD mice [73,74,75]. At the genetic level, mutant huntingtin drives iron overload by upregulating iron regulatory protein 1 (IRP1), and Berggren et al. found that brain iron accumulation in mutant huntingtin transgenic mice results from APP suppression [74,76]. Mechanistically, mutant HTT disrupts cellular iron handling and increases the expression of iron import proteins, thereby promoting iron accumulation in neuronal mitochondria. This mitochondrial iron overload directly leads to lipid ROS generation, linking iron to the energy deficits and oxidative damage characteristic of HD neurons [73,77]. Alongside iron dysregulation, lipid peroxidation and GSH depletion are prominent manifestations in HD. The plasma of HD patients shows high levels of lipid peroxidation and low levels of GSH, and these alterations are also present in the brains of HD animals, with oxidative stress occurring before the onset of HD symptoms [78]. The accumulation of toxic lipid peroxidation byproducts in the striatum is associated with worsening motor function, and ferroptosis is therefore considered a plausible explanation for the selective vulnerability of the striatum in HD. Treatment with ferroptosis inhibitors significantly ameliorates striatal cell death induced by 3-nitropropionic acid [79]. Figure 4 shows a schematic illustration of neuronal ferroptosis mechanisms and the vicious cycle in HD progression.

### 3.4. Ferroptosis and Amyotrophic Lateral Sclerosis (ALS)

Amyotrophic lateral sclerosis (ALS) is a progressive and fatal motor neuron disease that mainly affects motor neurons in the brain and spinal cord, and patients usually die of respiratory failure within 3 to 5 years after symptom onset [80,81]. The global pooled incidence of ALS is approximately 1.75 per 100,000 person-years, ranging from 0.73 to 1.89 per 100,000 person-years across regions. The mean age at onset is 55 to 65 years, and men are affected more often than women. Approximately 95% of cases are sporadic, whereas familial ALS accounts for only about 5% [82,83]. The involvement of ferroptosis in ALS is supported by several converging lines of evidence. Abnormal iron accumulation has been observed in multiple brain regions of ALS patients and animal models, including the spinal cord, motor cortex, substantia nigra, globus pallidus, red nucleus, and putamen, and the expression of multiple iron transport proteins (including DMT1, TFR1, FPN, and ceruloplasmin) is increased in the spinal cord of ALS animals [84,85]. A particularly consistent feature of ALS is GPX4 deficiency: GPX4 is absent in the postmortem spinal cord of ALS patients, and this is also a common early feature of SOD1G93A, TDP-43, and C9orf72 ALS mouse models [86]. Overexpression of GPX4 in SOD1G93A mice significantly delayed the onset of ALS, prolonged lifespan, and improved motor function [87]. Conversely, neuron-specific knockout of GPX4 leads to motor neuron degeneration, ALS-like paralysis, and death of spinal motor neurons [88]. Misfolded Cu/Zn superoxide dismutase 1 (SOD1) provides an additional toxic mechanism: it activates the myeloperoxidase/hypochlorous acid pathway, leading to GPX4 downregulation and promoting ferroptosis of motor neurons [89]. The ·OH generated by ferrous iron through the Fenton reaction is a major cause of neuronal injury in ALS [16]. Lipid peroxidation is likewise elevated in the blood of ALS patients, and ALS transgenic mice show GPX4 depletion and dysregulated GSH synthesis. Phospholipid peroxidation dependent on defects in the GSH/GPX4 axis can directly drive motor deficits in ALS [86]. Consistent with these mechanisms, edaravone, a clinically approved drug for ALS, inhibits ferroptosis by exerting antioxidant and lipid-stabilizing effects, thereby alleviating ALS [90]. Figure 5 shows a schematic illustration of neuronal ferroptosis mechanisms and the vicious cycle in ALS progression.

### 3.5. Ferroptosis and Multiple Sclerosis (MS)

Multiple sclerosis (MS) is the most common chronic inflammatory demyelinating disease of the central nervous system, characterized by myelin destruction, plaque formation, and astrogliosis [91,92]. MS predominantly affects young and middle-aged adults aged 20 to 40 years, with a mean age at diagnosis of approximately 32 years. The prevalence in women is about twice that in men, and the disease shows marked geographic variation, with significantly higher prevalence at high latitudes than at low latitudes. The number of people living with MS worldwide has reached approximately 2.8 million [93]. The role of ferroptosis in MS is reflected in several interrelated aspects, beginning with the close association between iron accumulation and demyelination. Iron is stored mainly in oligodendrocytes of the normal brain and participates in myelin formation, but iron concentrations are elevated in the brains of MS patients, particularly in gray matter and in the vicinity of MS lesions, which may be one cause of myelin loss in MS [94]. In the cuprizone-induced MS model, rapid iron mobilization from ferritin leads to ferroptosis, which in turn causes oligodendrocyte loss and iron-mediated lipid peroxidation and demyelination [95]. At the molecular level, reduced GPX4 expression together with elevated ACSL4 constitutes a ferroptotic signature of MS. In both MS and experimental autoimmune encephalomyelitis (EAE), the expression of the ferroptosis suppressor GPX4 is reduced, whereas ACSL4 content is significantly increased [96]. Targeted knockout of the ACSL4 gene can effectively inhibit the occurrence of ferroptosis [97]. Ferroptosis also engages in a vicious cycle with neuroinflammation. Ferroptotic neurons can activate T cells through TCR stimulation, leading to further deterioration of MS, and ferroptosis can induce neurodegeneration in MS by promoting T-cell activation [98]. The epigenetic regulator G9a can further determine neuronal susceptibility to inflammatory stress through transcriptional control of ferroptosis, further suggesting cross-regulation between ferroptosis and neuroinflammation [44]. In the peripheral nervous system, Schwann cells likewise depend on iron homeostasis: knockout of DMT1 and FTH in these cells reduces axonal proliferation, maturation, and myelination [99], underscoring the general dependence of myelinating glia on iron handling. Figure 6 shows a schematic illustration of neuronal ferroptosis mechanisms and the vicious cycle in MS progression. Finally, cell-type-specific responses should be considered: neurons, oligodendrocytes, astrocytes, and microglia differ markedly in iron handling, GPX4 dependence, and antioxidant reserves. Oligodendrocytes, which normally store iron for myelin synthesis, appear particularly vulnerable to ferroptosis in MS and other inflammatory demyelinating conditions [94,95,96], whereas microglial iron accumulation may amplify neuroinflammation. These differences have implications for the design of cell-type-targeted anti-ferroptotic interventions (Figure 6).

### 3.6. Ferroptosis in Other Central Nervous System Disorders: Complementary Evidence Beyond the Core Scope

Beyond the five neurodegenerative diseases that constitute the core scope of this review, ferroptosis has also been implicated in other central nervous system (CNS) disorders, including cerebral ischemia–reperfusion injury, intracerebral hemorrhage, spinal cord injury, and depression [61,100,101]. These conditions share the core ferroptotic features of iron accumulation, glutathione depletion, and lipid peroxidation, and several TCM interventions discussed in Section 4, such as electroacupuncture at Shenmen (HT7) and Dazhui (GV14) in cerebral hemorrhage models and eye-acupuncture in cerebral ischemia–reperfusion models, were investigated in these indications [102,103]. Because these disorders are acute or non-degenerative CNS conditions rather than classical neurodegenerative diseases, the corresponding studies are classified as complementary evidence and excluded from the core neurodegenerative-disease efficacy evidence; they illustrate the broader anti-ferroptotic pharmacology of TCM in the CNS. Likewise, entries involving glioma cells, cancer models, cuproptosis, or synthetic TCM-inspired hybrid molecules (Table 2, Table 7 and Table 12) are marked as Complementary, whereas rows combining core and non-core indications are marked as Mixed; neither category is counted as core neurodegenerative evidence.

In summary, ferroptosis has been implicated in the pathological progression of five neurodegenerative diseases, namely AD, PD, HD, ALS, and MS. In each of these diseases, neuronal injury converges on the shared execution and defense modules detailed in Section 2, while mitochondrial involvement remains context-dependent. Although these diseases differ in etiology and pathological features, they all present hallmark ferroptotic changes such as abnormal brain iron deposition, elevated lipid peroxidation, and reduced antioxidant defense capacity, supporting ferroptosis as a convergent mechanism associated with neuronal death across these diseases. Across these five diseases, iron accumulation and failure of the GPX4/GSH system recur as shared downstream nodes, whereas the upstream triggers are disease-specific, such as Aβ/Tau pathology in AD, α-synuclein in PD, mutant huntingtin in HD, mutant SOD1 in ALS, and inflammatory demyelination in MS. This pattern of convergent execution downstream of divergent triggers provides the mechanistic rationale for the intervention strategies discussed in the next section.

## 4. Traditional Chinese Medicine Targeting Ferroptosis for the Prevention and Treatment of Neurodegenerative DISEASES: Bioactive Components, Compound Formulas, and Characteristic TCM Therapies

The material basis of TCM for regulating ferroptosis encompasses bioactive components of individual herbs (flavonoids, saponins, phenolics, polysaccharides, terpenoids, and lignans), multi-component pharmacological networks of compound formulas, and physical stimulation therapies such as acupuncture, moxibustion, and electroacupuncture. The underlying mechanisms involve multidimensional regulation of iron metabolism, antioxidant systems such as GPX4, key enzymes of lipid metabolism such as ACSL4, and nuclear transcription factors such as Nrf2 and p53. Together, these categories provide the organizational framework used below to evaluate ferroptosis-related TCM evidence [104,105].

Throughout this section, a distinction is maintained between purified, chemically defined compounds (Section 4.1) and complex herbal formulations such as multi-herb compound formulas and decoctions (Section 4.2). These two categories differ fundamentally in reproducibility and pharmacological interpretation: purified compounds permit defined dose–exposure–response relationships and unambiguous mechanistic attribution, whereas formulations are mixtures of constituents whose exposure, interactions, and batch consistency are difficult to control. Mechanistic conclusions drawn from formulations should therefore be regarded as network-level associations unless the active constituents have been identified and individually validated.

### 4.1. Bioactive Components of Individual Herbs: Key Tools Targeting the Core Pathways of Ferroptosis

Bioactive compounds isolated from individual herbs provide chemically defined candidates for testing ferroptosis-related mechanisms. In models of Alzheimer’s disease (AD) and Parkinson’s disease (PD), their reported effects converge mainly on the Nrf2/GPX4 antioxidant axis, iron-handling proteins (TFR1/FPN1/FTH1/NCOA4), and ACSL4-mediated phospholipid peroxidation. These findings identify candidate intervention nodes, but the strength of mechanistic attribution depends on the rescue and genetic-validation criteria summarized in Section Literature Identification and Scope and Table 1, Table 2, Table 3, Table 4, Table 5, Table 6, Table 7, Table 8, Table 9, Table 10, Table 11 and Table 12.

#### 4.1.1. Flavonoids

Flavonoids are extensively studied modulators of ferroptosis in neurodegenerative diseases. Eriodictyol mediates the Nrf2/HO-1 pathway through the vitamin D receptor, upregulates GPX4 and the iron exporter FPN1, downregulates TFRC and FTH, restores iron metabolic balance, and inhibits lipid peroxidation in APP/PS1 mice [106]. Quercetin upregulates GPX4/SLC7A11 and downregulates NCOA4 through Nrf2 signaling, thereby antagonizing MPP^+^-induced ferroptosis of dopaminergic neurons [107]. Myricetin alleviates motor impairment in MPTP rats through the Nrf2/GPX4 pathway [108]. Herbacetin activates the Nrf2/GPX4 pathway and inhibits ferroptosis induced by 6-OHDA and RSL3 [109]. In a streptozotocin (STZ)-induced sporadic AD model, silibinin inhibits p53, upregulates SLC7A11/GPX4, and alleviates ferroptotic damage, thereby blocking STING-mediated neuroinflammation induced by oxidative DNA leakage and forming a novel AD pathogenic loop of lipid peroxidation, DNA damage, and innate immune activation [110]. Total flavonoids of Astragalus (TFA) likewise inhibit ferroptosis by regulating the SLC7A11/GPX4 axis and exert neuroprotective effects against MPTP/MPP^+^-induced neurodegeneration [111]. Icariin improves cognition in APP/PS1 mice through the MDM2-p53-SLC7A11 axis [112], and a mixture of icariin, astragaloside IV, and puerarin has been reported to exert combined anti-ferroptotic effects through the System Xc^−^-GPX4-ACSL4-LPCAT3 axis [113]. Fan et al. found that icariside II (ICS II) directly binds Keap1 and activates the Keap1/Nrf2/GPX4 signaling axis, upregulating the expression of Nrf2, heme oxygenase-1 (HO-1), and GPX4 while reducing Keap1 protein levels [114]. ICS II simultaneously suppresses apoptosis, oxidative stress, and ferroptosis in PD cell models, which supports it as a candidate molecule for ferroptosis-targeted intervention in PD. Baicalein binds to and protects aconitase 1 (ACO1) from ROS/RNS-induced oxidative inactivation, thereby inhibiting its pathological conversion to iron regulatory protein 1 (IRP1), which downregulates transferrin receptor 1 and divalent metal transporter 1 and upregulates ferroportin 1, ultimately attenuating brain iron accumulation and iron-dependent oxidative stress in a rotenone-induced PD model [115]. Baicalein also improves motor and cognitive dysfunction and promotes remyelination in a multiple sclerosis (MS) model through antioxidant mechanisms [116], and it alleviates neuroinflammation in MPTP-induced PD mice by inhibiting the NLRP3/caspase-1/GSDMD pathway [117]. In ALS models, kaempferol (KP) counteracts neurotoxicity by reducing the intracellular accumulation of mutant SOD1 protein and inhibiting mutant SOD1-induced mitochondrial superoxide production [118,119]. Wang et al. demonstrated that, compared with wild-type SOD1 fibrils, amyloid fibril structures of SOD1 cause marked mitochondrial damage and activate ferroptosis in cell culture [120]. Epigallocatechin gallate (EGCG) is the most abundant and biologically active characteristic component of tea (Camellia sinensis) [121]. Clinical evidence shows that Nrf2 protein levels in the motor cortex and spinal cord of ALS patients are significantly downregulated compared with controls [122], whereas EGCG upregulates Nrf2 and GPX4 expression in iron-overloaded mice, enhances overall antioxidant capacity, and clearly exhibits ferroptosis-inhibiting activity [123]. Beyond neurological disease models, EGCG has been shown to modulate ferroptosis in cancer cells through tsRNA-mediated regulation [124] and to inhibit iron deposition and apoptosis in rats with cerebral hemorrhage [100]. Given that skeletal muscle iron accumulation has been reported in ALS patients, the ferroptosis-modulating activity of EGCG warrants direct investigation in ALS models. Xia et al. confirmed in a Drosophila PD model that inhibition of ferroptosis is an important mechanism underlying the neuroprotective effects of EGCG [125]. Reznichenko et al. further investigated the regulatory effects of EGCG on the iron metabolism-related proteins APP and transferrin receptor (TfR) and confirmed the regulatory efficacy of EGCG in AD [126]. Wang et al. showed that in the hSOD1G93A transgenic mouse model, fisetin improves motor performance, delays disease onset, and increases spinal motor neuron density, with antioxidant and neuroprotective effects both in vivo and in vitro. Mechanistically, these effects are accompanied by decreased ROS levels, upregulated expression of phosphorylated extracellular signal-regulated kinase (p-ERK), and elevated levels of endogenous antioxidant molecules [127]. Further studies have shown that fisetin can increase GSH concentration by promoting cysteine uptake and enhancing glutamate-cysteine ligase activity, thereby strengthening cellular antioxidant reserves. It also exerts anti-inflammatory regulation on microglia by inhibiting 5-lipoxygenase (5-LOX) activity, reducing the release of pro-inflammatory cytokines, and decreasing the production of lipid peroxidation products [128]. Recent mechanistic studies report that fisetin improves cell survival and morphological damage by inhibiting ferroptosis and oxidative stress, while simultaneously reducing ROS generation and lipid peroxidation [129]. In summary, flavonoids provide multi-target candidate compounds for further evaluation in neurodegenerative disease models (Table 1).

**Table 1:** Mechanisms of flavonoids in regulating ferroptosis.

Component	Source	Disease	Model	Signaling Pathway	Key Mechanisms	Ref./ Level
Eriodictyol	Citrus peels and Chinese herbs	AD	APP/PS1 mice	VDR-mediated Nrf2/HO-1	GPX4 ↑, VDR ↑, p-Nrf2 ↑, FPN1 ↑; TFRC ↓, FTH ↓, Aβ/p-Tau ↓, Fe^2+^/MDA/ROS ↓	[106] D
			HT-22 cells (Aβ1-42-induced)			
Quercetin	Widely distributed in plants	PD	SH-SY5Y cells (MPP^+^); primary cortical neurons (MPP^+^)	Nrf2 signaling	GPX4 ↑, Nrf2 ↑, SLC7A11 ↑, MMP ↑; NCOA4 ↓, MDA ↓, iron ↓, lipid peroxidation↓	[107] I
Myricetin	Flavonoid (numerous plants)	PD	MPTP rats	Nrf2/GPX4 (promotes Nrf2 nuclear translocation)	Fe^2+^ ↓, ROS ↓, MDA ↓, GSH ↑, TH ↑, α-Syn ↓	[108] S
			SH-SY5Y cells (MPP^+^)			
Herbacetin	Rhodiola/Gossypium species	PD	PC12 cells (6-OHDA- and RSL3-induced)	Nrf2/GPX4	GPX4 ↑, SLC7A11 ↑, HO-1 ↑, NQO1 ↑, GSH ↑; MDA ↓, ROS ↓, lipid peroxidation ↓	[109] D
Silibinin	*Silybum marianum*	AD (sporadic)	ICV-STZ rats	p53-SLC7A11/GPX4, STING	p53 ↓, SLC7A11 ↑, GPX4 ↑; oxidative DNA leakage ↓, STING ↓, inflammatory cascade ↓	[110] S
			STZ-treated HT-22 cells			
Total flavonoids of Astragalus (TFA)	*Astragalus membranaceus*	PD	MPTP mice	SLC7A11/GPX4	SLC7A11 ↑, GPX4 ↑, GSH ↑	[111] I
			SH-SY5Y cells (MPP^+^)			
Icariin	*Epimedium brevicornu*	AD	APP/PS1 mice	MDM2→p53/SLC7A11	SOD ↑, JC-1 aggregate ratio ↑; brain iron ↓, MDA ↓, mitochondrial lipid peroxidation ↓	[112] I
Icariside II (ICS II)	*Epimedium brevicornu*	PD	SK-N-SH cells (MPP^+^)	Keap1/Nrf2/GPX4	Keap1 ↓, Nrf2 ↑, HO-1 ↑	[114] I
Baicalein	*Scutellaria baicalensis*	PD; MS	PD: rotenone-induced rats and MPTP-induced mice; MS: cuprizone-fed mice	ACO1 protection/iron metabolism, antioxidant, NLRP3/caspase-1/GSDMD	Brain iron accumulation ↓, iron-dependent oxidative stress ↓; remyelination ↑; neuroinflammation ↓	[115,116,117] M
			MS: cultured oligodendrocyte-lineage cells and OLN-93 cells			
Kaempferol	Various plants	ALS	N2a cells overexpressing mutant SOD1	Antioxidant (counteracting mutant SOD1 toxicity)	Mutant SOD1 accumulation ↓, mitochondrial superoxide ↓	[118,119] I

Note: ↑, upregulated or increased; ↓, downregulated or decreased; →, direction of effect. Evidence levels are defined in Section Literature Identification and Scope: D, Direct; S, Supportive; I, Indirect; M, Mixed; C, Complementary or outside the core scope.

#### 4.1.2. Glycosides

Glycosides regulate neurodegenerative ferroptosis through multiple pathways. Salidroside is the most extensively studied representative. In AD, it inhibits Aβ1-42- and glutamate-induced ferroptosis through the Nrf2/HO-1 and Nrf2/GPX4 axes and the VDAC1-AKT/GSK-3β pathway [130,131,132], and in PD it antagonizes MPTP-induced ferroptosis and improves motor function through the Nrf2/GPX4 pathway [133]. Tetrahydroxystilbene glycoside (TSG) activates the Keap1/Nrf2/ARE and GSH/GPX4/ROS pathways, downregulates DMT1, ACSL4, and NCOA4, and reduces Aβ deposition [134]. Paeoniflorin inhibits neuronal ferroptosis in AD mice through the p53-SLC7A11 axis [135]. Morroniside and sennoside A improve PD and AD through the Nrf2/ARE and TRAF6-NF-κB pathways, respectively [136,137]. Astragalus saponins restore cerebral iron homeostasis and lipid peroxidation balance through the NOX4/Nrf2 pathway [138], and the icariin-astragaloside IV-puerarin mixture inhibits ferroptotic lipid peroxidation through the System Xc^−^-GPX4-ACSL4-LPCAT3 axis [113]. Gastrodin inhibits H_2_O_2_-induced ferroptosis in C6 glioma cells through its antioxidant effects and upregulates Nrf2/GPX4 and FPN1 [139]. The phenylethanoid glycosides forsythoside A and isoforsythiaside and the phenolic glycoside curculigoside ameliorate AD pathology through the Nrf2/GPX4 and GPX4/SLC7A11 pathways, respectively [140,141,142]. Acteoside (verbascoside) improves PD through Nrf2-mediated mitophagy and the Nrf2/SLC7A11/GPX4 pathway [143,144]. Polygala-related components (tenuifolin and senegenin) protect hippocampal neurons through the SLC7A11/GSH/GPX4 and ACSL4/PEBP1/GPX4 pathways [145,146]. In summary, glycosides exert neuroprotective effects by coordinately regulating ferroptosis, oxidative stress, and iron metabolism (Table 2).

**Table 2:** Mechanisms of glycosides (including phenylethanoid glycosides, phenolic glycosides, and saponins) in regulating ferroptosis.

Component	Source	Disease	Model	Signaling Pathway	Key Mechanisms	Ref./ Level
Salidroside	*Rhodiola rosea*	AD; PD	AD: Aβ1-42-injured mice, SAMP8 mice; PD: MPTP mice	Nrf2/GPX4, Nrf2/HO-1, VDAC1-AKT/GSK-3β	GPX4 ↑, SLC7A11 ↑, Nrf2 ↑, GSH ↑, MMP ↑; Fe^2+^ ↓, ACSL4 ↓, TFR1 ↓, MDA ↓, α-syn ↓	[130,131,132,133] M
			AD: SH-SY5Y cells (Aβ1-42)			
Tetrahydroxystilbene glycoside (TSG)	*Polygonum multiflorum*	AD	APP/PS1 mice	Keap1/Nrf2/ARE, GSH/GPX4/ROS	GPX4 ↑, GPX1 ↑, xCT ↑, CD98 ↑, FTH1 ↑, Nrf2 ↑; Aβ ↓, DMT1 ↓, ACSL4 ↓, NCOA4 ↓, NLRP3 ↓	[134] I
Paeoniflorin	*Paeonia lactiflora Pall*./*Paeonia suffruticosa*	AD	APP/PS1 mice	p53/SLC7A11	GPX4 ↑, SLC7A11 ↑, SOD ↑, JC-1 ↑; p53 ↓, iron ↓, MDA ↓, ROS ↓	[135] I
			PC12 cells (erastin-induced)			
Morroniside	*Cornus officinalis*	PD	MPTP mice	Nrf2/ARE	GSH ↑, GPX4 ↑, SLC7A11 ↑, FTH1 ↑, FPN ↑; MDA ↓, iron ↓, ROS ↓	[136] I
			PC12 cells (MPP^+^)			
Sennoside A	*Rheum palmatum*	AD	APP/PS1 mice	TRAF6-NF-κB	Hippocampal Fe^2+^ ↓, MDA ↓, ROS ↓, GSH ↑; inflammatory factors ↓, caspase-3 ↓	[137] I
			BV-2 microglia (LPS-induced)			
Astragaloside IV / Astragalus saponins	*Astragalus membranaceus*	AD	SAMP8 mice; APP/PS1 mice	NOX4/Nrf2	GPX4 ↑, SLC7A11 ↑, FTH1 ↑, FPN1 ↑; NOX4 ↓, Fe^2+^ ↓, MDA ↓	[113,138] M
Gastrodin	*Gastrodia elata*	Glioma (complementary evidence; outside core ND scope)	Rat C6 glioma cells (H_2_O_2_-induced)	Nrf2/GPX4	Nrf2 ↑, GPX4 ↑, FPN1 ↑, HO-1 ↑	[139] C
Forsythoside A	*Forsythia suspensa*	AD	APP/PS1 mice	Nrf2/GPX4 + inhibition of IKK/NF-κB	GPX4 ↑, Nrf2 ↑, xCT ↑, FTH/FTL/FPN1 ↑, GSH ↑; MDA ↓, TFRC ↓, Aβ/p-tau ↓, inflammation ↓	[140] I
			N2a, HT-22 and BV-2 cells			
Isoforsythiaside	*Forsythia suspensa*	AD	APP/PS1 mice	Nrf2	GPX4 ↑, Nrf2 ↑, p-GSK-3β ↑; TFR1 ↓, DMT1 ↓	[141] I
			HT-22 cells			
Curculigoside	*Curculigo orchioides*	AD	Scopolamine- and okadaic acid-induced ICR mice	GPX4/SLC7A11, p-BECN1	GPX4 ↑, GCLM/GCLC ↑, FPN1 ↑, GSH/GSSG ↑; ACSL4 ↓, p-tau ↓, Aβ ↓, iron/MDA/ROS ↓	[142] I
			SH-SY5Y cells			
Acteoside (verbascoside)	*Cistanche deserticola*	PD	MPTP mice	Nrf2-mediated mitophagy (PINK1/Parkin), Nrf2/SLC7A11/GPX4	GPX4 ↑, SLC7A11 ↑; mitoROS ↓, MDA ↓, iron ↓	[143,144] M
			SH-SY5Y cells (erastin-, MPP^+^- or salsolinol-induced)			
Tenuifolin	*Polygala tenuifolia*	AD	D-galactose plus Aβ1-42 mice	calpain + SLC7A11/GSH/GPX4 + BDNF/TrkB	GPX4 ↑, SLC7A11 ↑, GSH ↑, BDNF ↑, PSD95 ↑; Ca^2+^ ↓, m-calpain ↓, ACSL4 ↓, MDA ↓	[145] I
			HT-22 cells			
Senegenin	*Polygala tenuifolia*	AD	PC12 cells (Aβ25-35-induced)	Lipid metabolism/ferroptosis (ACSL4, PEBP1, GPX4)	GPX4 ↑, GPx activity ↑; ACSL4 ↓, PEBP1 ↓, ROS ↓, MDA ↓	[146] S

Note: ↑, upregulated or increased; ↓, downregulated or decreased; →, direction of effect. Evidence levels are defined in Section Literature Identification and Scope: D, Direct; S, Supportive; I, Indirect; M, Mixed; C, Complementary or outside the core scope.

#### 4.1.3. Phenolic Compounds

Phenolic compounds inhibit ferroptosis mainly through the Nrf2 pathway and downstream antioxidant effects. Tannic acid enhances GPX4 activity and expression, promotes Nrf2 nuclear translocation, and chelates Fe^3+^, thereby antagonizing the aggregation of Aβ42 and tau as well as lipid peroxidation [147,148]. Curcumin has strong metal-chelating activity, antagonizes Fe^3+^/H_2_O_2_-induced oxidative damage, and inhibits Aβ/tau aggregation and NF-κB-mediated neuroinflammation [7,149]. Resveratrol protects SLC7A11 through SIRT1/p53 and activates the SIRT3/FoxO3a antioxidant axis, showing anti-ferroptotic activity in ALS models [150]. Alpha-lipoic acid, an endogenous disulfide compound rather than a plant polyphenol, regulates iron metabolism through the SIRT1/Nrf2 pathway (FPN ↑, FTH1 ↑, DMT1 ↓) [151]. Corilagin attenuates ferroptosis and apoptosis of dopaminergic neurons in MPTP mice through the TLR4/Src/NOX2 pathway [152]. Ferulic acid inhibits copper-induced ferroptosis through the SIRT1/GPX4 pathway and improves cognitive impairment associated with Wilson’s disease (hepatolenticular degeneration) [153]. The multi-target properties of phenolic compounds make them candidate agents combining antioxidant, iron-chelating, and anti-inflammatory activities (Table 3).

**Table 3:** Mechanisms of phenolic compounds in regulating ferroptosis.

Component	Source	Disease	Model	Signaling Pathway	Key Mechanisms	Ref./ Level
Tannic acid	Natural polyphenol (pentagalloyl glucose)	AD	SH-SY5Y cells (RSL3- and Aβ42-induced)	GPX4 enhancement, Nrf2-GPX4, Fe chelation	GPX4 activity/level ↑, Nrf2 nuclear translocation ↑; LIP ↓, mitoROS ↓, Aβ42/tau aggregation ↓, lipid peroxidation ↓	[147,148] M
Curcumin	*Curcuma longa*	AD	N2a and HEK293 cells	Metal chelation, anti-aggregation, NF-κB inhibition	Fe^3+^ chelation counteracts oxidative damage; Aβ/tau aggregation ↓, neuroinflammation ↓	[7,149] I
Resveratrol	*Vitis vinifera*/red wine	ALS	SOD1G93A mice	SIRT1/p53→SLC7A11, SIRT3/FoxO3a→SOD2	GPX4 ↑, SLC7A11 ↑, SOD2 ↑, GSH balance ↑; Fe^2+^ ↓, lipid peroxidation ↓	[150] I
			HT-22 cells			
Alpha-lipoic acid	Endogenous disulfide compound	PD	MPTP mice	SIRT1/Nrf2	FPN ↑, FTH1 ↑, GPX4 ↑, xCT ↑, GSH ↑; DMT1 ↓, Fe^2+^ ↓, ROS ↓, lipid peroxidation ↓	[151] I
			PC12 cells (6-OHDA-induced)			
Corilagin	Tannins	PD	MPTP mice	TLR4/Src/NOX2	GPX4 ↑, SOD ↑, CAT ↑, GSH-Px ↑; ROS ↓, iron ↓, MDA ↓, TH neurons ↑	[152] I
			N2a cells (MPP^+^)			
Ferulic acid	*Ligusticum chuanxiong*/*Angelica sinensis*, etc.	Wilson’s disease	Copper-loaded WD rats	SIRT1/GPX4	GPX4 ↑, inhibition of Cu^2+^-induced ferroptosis	[153] C
			copper-overloaded PC12 cells			

Note: ↑, upregulated or increased; ↓, downregulated or decreased; →, direction of effect. Evidence levels are defined in Section Literature Identification and Scope: D, Direct; S, Supportive; I, Indirect; M, Mixed; C, Complementary or outside the core scope.

#### 4.1.4. Polysaccharides

Polysaccharides have been evaluated as modulators of ferroptosis-related pathways in neurological disease models. Wolfberry glycopeptide reduces the labile iron pool and lipid peroxidation by regulating iron metabolism (TFRC ↓, FTH1 ↑, FPN ↑) and restores dopaminergic neurons in PD mice, and its effect is independent of GPX4 [154]. A neutral polysaccharide from Gastrodia elata inhibits ferroptosis-mediated neuroinflammation through the Nrf2/HO-1 pathway and alleviates cerebral ischemia–reperfusion injury [155]. Polysaccharides mainly exert anti-ferroptotic activity through the Nrf2/GPX4 axis or iron homeostasis regulation (Table 4).

**Table 4:** Mechanisms of polysaccharides in regulating ferroptosis.

Component	Source	Disease	Model	Signaling Pathway	Key Mechanisms	Ref./ Level
Wolfberry (Lycium barbarum) glycopeptide	*Lycium barbarum*	PD	SNCA (A53T) mice; MPTP mice; 6-OHDA rats	Iron homeostasis regulation (GPX4-independent)	TFRC ↓, FTH1 ↑, FPN ↑ → LIP ↓, Fe^2+^ ↓; 4-HNE ↓, MDA ↓, GSH ↑; TH/DAT ↑, inflammation ↓	[154] S
			PC12 cells			
Gastrodia elata neutral polysaccharide	*Gastrodia elata*	Cerebral ischemia–reperfusion injury	HT-22 cells (OGD/R)	Nrf2/HO-1	Inhibition of ferroptosis-mediated neuroinflammation, antioxidant	[155] C

Note: ↑, upregulated or increased; ↓, downregulated or decreased; →, direction of effect. Evidence levels are defined in Section Literature Identification and Scope: D, Direct; S, Supportive; I, Indirect; M, Mixed; C, Complementary or outside the core scope.

#### 4.1.5. Terpenoids

Terpenoids exert anti-ferroptotic effects in neurodegenerative diseases through multiple pathways. Artemisinin competitively binds KEAP1, promotes KEAP1-Nrf2 dissociation, and inhibits Nrf2 ubiquitination, thereby activating the SLC7A11/GPX4 pathway and antagonizing erastin- and glutamate-induced ferroptosis in AD models [156]. Ganoderic acid A inhibits ferroptosis and lipid peroxidation in AD through the Nrf2/SLC7A11/GPX4 pathway [157] and exerts protective effects in PD by inhibiting NCOA4-mediated ferritinophagy [158]. Pachymic acid improves cognitive impairment in AD rats through the Nrf2/SLC7A11/GPX4 pathway [159]. Buddlejasaponin IVb ameliorates PD by inhibiting IRP2-mediated iron overload, and its anti-ferroptotic effect depends mainly on iron reduction rather than restoration of GPX4/GSH [160]. Saikosaponin B2 ameliorates microglial activation in depression models by inhibiting ferroptosis-mediated neuroinflammation and endoplasmic reticulum stress [161], and celastrol improves PD-associated ferroptosis through the Nrf2-NLRP3-caspase-1 axis [162]. Ginsenosides (Rg1/Rg3/Rb1/Re) maintain DMT1/FPN1 iron homeostasis and activate the Nrf2/xCT/GPX4 axis, thereby interrupting the vicious cycle of iron deposition, oxidative stress, and lipid peroxidation [163]. The sesquiterpene lactone OABL exerts anti-ferroptotic and anti-neuroinflammatory effects through GSH-Michael addition [164]. Ginkgolide B inhibits Aβ1-42-induced ferroptosis and oxidative stress in N2a cells by upregulating FTH1 and downregulating SPP1 [165]. Reported terpenoid effects converge on Nrf2 signaling, iron homeostasis, and mitochondrial regulation (Table 5).

**Table 5:** Mechanisms of terpenoids in regulating ferroptosis.

Component	Source	Disease	Model	Signaling Pathway	Key Mechanisms	Ref./ Level
Artemisinin	*Artemisia annua*	AD	3×Tg mice	KEAP1-Nrf2-SLC7A11-GPX4	Competitive binding to KEAP1, promoting Nrf2 nuclear translocation; SLC7A11 ↑, GPX4 ↑, GSH ↑, MDA ↓	[156] D
			HT-22 and SH-SY5Y cells (erastin/glutamate-induced)			
Ganoderic acid A	*Ganoderma lucidum*	AD	APP/PS1 mice	Nrf2/SLC7A11/GPX4	Nrf2 ↑, SLC7A11 ↑, GPX4 ↑; MDA ↓, ROS ↓, lipid peroxidation ↓	[157] I
			HT-22 cells (Aβ25-35-induced)			
Ganoderic acid A	*Ganoderma lucidum*	PD	MPTP mice	NCOA4 ferritinophagy	NCOA4 ↓, LC3B ↓, FTH1 ↑, p62 ↑, iron ↓	[158] D
			SH-SY5Y cells and primary midbrain neurons (MPP^+^)			
Pachymic acid	*Poria cocos*	AD	Aβ25-35-injected rats	Nrf2/SLC7A11/GPX4	Nrf2 ↑, SLC7A11 ↑, GPX4 ↑, GSH ↑; MDA ↓, Fe^2+^ ↓, iron deposition ↓	[159] I
Buddlejasaponin IVb	*Clinopodium chinense*	PD	MPTP mice	IRP2 iron transport	Inhibition of IRP2, alleviation of iron overload; iron ↓, MDA ↓, 4-HNE ↓, TFR ↓, FPN1 ↑, FTH ↑, TH neurons ↑ (GPX4/GSH not restored)	[160] D
			SH-SY5Y cells and primary midbrain neurons (MPP^+^)			
Saikosaponin B2, Celastrol	*Bupleurum chinense*/*Tripterygium wilfordii*	Depression; PD	Depression: CUMS mice; PD: MPTP mice and AAV-α-synuclein-overexpressing mice	TLR4/NF-κB, Nrf2	Saikosaponin B2: inhibition of LPS-induced microglial activation and ferroptosis; Celastrol: iron/ROS reduction, upregulation of HO-1/NQO1	[161,162] M
			Depression: LPS-induced primary microglia			
Ginsenosides (Rg1/Rg3/Rb1/Re, etc.)	*Panax ginseng*	PD	MPTP-, 6-OHDA- and rotenone-induced PD models	Nrf2/xCT/GPX4, DMT1/FPN1 iron homeostasis, PINK1/Parkin	GPX4 ↑, DMT1 ↓, FPN1 ↑ → LIP ↓, α-syn fibrillation ↓, mitophagy ↑	[163] I
OABL (1,6-O,O-diacetylbritannilactone)	*Inula japonica*	AD	5xFAD mice	GSH-Michael addition, NF-κB	Resistance to RSL3-induced ferroptosis (survival 17.1% → 70.8%), Aβ ↓, p-Tau ↓, BACE1 ↓, GSH ↑, MDA ↓	[164] I
			BV-2 and PC12 cells			
Ginkgolide B (diterpene lactone)	*Ginkgo biloba*	AD	N2a cells (Aβ1-42-induced)	Ferroptosis-oxidative stress pathway (FTH1 ↑/SPP1 ↓)	FTH1 ↑, SPP1 ↓, SOD ↑; ROS ↓, MDA ↓, Aβ ↓, anti-apoptosis (Bax ↓/Bcl-2 ↑)	[165] I

Note: ↑, upregulated or increased; ↓, downregulated or decreased; →, direction of effect. Evidence levels are defined in Section Literature Identification and Scope: D, Direct; S, Supportive; I, Indirect; M, Mixed; C, Complementary or outside the core scope.

#### 4.1.6. Lignans

Lignans regulate ferroptosis through specific molecular mechanisms. Schisandrin B inhibits GSK3β activation, suppresses neuronal ferroptosis through the Nrf2/GPX4 pathway with reported cooperation from FSP1-dependent defense, and thereby blocks the release of TNF-α from ferroptotic neurons and inhibits microglial M1 polarization, improving AD pathology in 3×Tg mice [166]. Total Schisandra lignans restore cerebral iron homeostasis through the Nrf2/FPN1 signaling pathway, downregulate ACSL4, TFR, and DMT1, and improve cognition in SAMP8 mice [167]. Reported lignan effects converge on GSK3β/Nrf2/GPX4 signaling and FPN1-mediated iron export (Table 6).

**Table 6:** Mechanisms of lignans in regulating ferroptosis.

Component	Source	Disease	Model	Signaling Pathway	Key Mechanisms	Ref./ Level
Schisandrin B	*Schisandra chinensis*	AD	3×Tg mice	GSK3β/Nrf2/GPX4 + FSP1	Inhibition of GSK3β, Nrf2/GPX4 ↑, FSP1 ↑; lipid peroxidation ↓, MDA ↓, Fe^2+^ ↓, 4-HNE ↓, M1 polarization ↓	[166] S
			SH-SY5Y/APP695swe cells (erastin-induced); BV-2 microglia			
Total Schisandra lignans	*Schisandra chinensis*	AD	SAMP8 mice	Nrf2/FPN1	Nuclear Nrf2 ↑, FPN1 ↑, SLC7A11 ↑, GPX4 ↑; ACSL4 ↓, TFR ↓, DMT1 ↓, Fe^2+^/total iron ↓, p-Tau ↓	[167] I
			HT-22 cells			

Note: ↑, upregulated or increased; ↓, downregulated or decreased; →, direction of effect. Evidence levels are defined in Section Literature Identification and Scope: D, Direct; S, Supportive; I, Indirect; M, Mixed; C, Complementary or outside the core scope.

#### 4.1.7. Other Structural Classes

Beyond classic natural products, other compounds with distinctive structures also show potential against neurodegenerative ferroptosis. The anthraquinone emodin inhibits ferroptosis of dopaminergic neurons in PD by activating the mitochondrial complex III subunit UQCRC1 [168], and chrysophanol improves cognition in AD through the GSH/GPX4 system [169]. The tropolone hinokitiol inhibits ferroptosis in AD cells through the Nrf2 pathway and iron chelation [170]. Berberine inhibits JNK-p38MAPK and activates autophagy, downregulates ferroptosis-related genes (TFR1/ACSL4/DMT1/IREB2), and improves AD pathology in 5xFAD mice [171]. Muscone directly targets GSK-3β, restores the System Xc^−^-GSH-GPX4 axis, and regulates iron metabolism, with anti-ferroptotic efficacy comparable to that of Fer-1 and DFO [172]. Cyclovirobuxine D (an alkaloid) inhibits glutamate-induced ferroptosis in SH-SY5Y cells through Nrf2 and liquid–liquid phase separation of FTH1 [173]. Thonningianin A directly binds to and activates GPX4 through the AMPK/Nrf2/GPX4 pathway and chelates iron, thereby ameliorating AD [174]. Syringin stabilizes Nrf2 by enhancing the iASPP-Keap1 interaction, enhances synaptic plasticity, and improves cognitive impairment in AD models [175]. A water extract of Moschus (WEM) restores iron homeostasis through the Keap1/Nrf2 pathway [176]. Synthetic polycatechols (PDP/PLDP) inhibit ferroptosis through Fe^3+^ chelation, sequestration of the labile iron pool, and modulation of tau liquid–liquid phase separation [177]. The gallic acid-tryptophan cyclodipeptide hybrid molecule (GCTR) combines Fe^3+^ chelation, GPX4 activity enhancement, and modulation of tau liquid–liquid phase separation, concurrently attenuating ferroptosis and amyloid-associated toxicity [178]. These natural and synthetic products illustrate the structural diversity of reported ferroptosis-modulating agents (Table 7).

**Table 7:** Mechanisms of compounds with other structural classes in regulating ferroptosis.

Component	Source	Disease	Model	Signaling Pathway	Key Mechanisms	Ref./ Level
Emodin (anthraquinone)	*Polygonum cuspidtum*/*Rheum palmatu*	PD	PC12 cells (MPP^+^- and erastin-induced)	Mitochondrial complex III subunit UQCRC1	UQCRC1 ↑, GPX4 ↑, FTH ↑, SLC7A11 ↑; mitochondrial Fe^2+^/ROS ↓, MMP ↑, fragmentation ↓	[168] I
Chrysophanol (anthraquinone)	*Rheum palmatum*	AD	D-galactose plus Aβ25-35 rats	GSH/GPX4 system	GPX4 ↑, GPx activity ↑, GSH ↑; ROS ↓, LPO/MDA ↓, cognition ↑	[169] I
			PC12 cells			
Hinokitiol (tropolone)	*Chamaecyparis*	AD	zebrafish; mice	Nrf2, iron chelation	GPX4 ↑, SLC7A11 ↑, HO-1 ↑	[170] I
			PC12 cells			
Berberine	*Coptis chinensis*	AD	5xFAD mice	JNK-p38MAPK inhibition, autophagy	Autophagy ↑ (Beclin1 ↑, LC3B ↑, P62 ↓); TFR1/ACSL4/DMT1/IREB2 ↓, FTH1 ↑, SLC7A11 ↑, Aβ ↓, ROS ↓, MDA ↓	[171] I
			SH-SY5Y cells (Aβ-induced)			
Muscone	Natural musk	PD	MPTP mice	Inhibition of GSK-3β	p-GSK-3β ↑, GPX4 ↑, SLC7A11 ↑, FTH1 ↑, FPN ↑; Fe^2+^ ↓, ROS ↓, MDA ↓, TH ↑	[172] D
			MN9D cells			
Cyclovirobuxine D	*Buxus* (boxwood)	AD	SH-SY5Y cells (glutamate- and erastin-induced)	Nrf2, liquid–liquid phase separation of FTH1	Nuclear Nrf2 ↑, FTH1 ↑, GPX4 ↑, GSH ↑; Fe^2+^ ↓, iron deposition ↓, MDA ↓, mitochondrial protection	[173] I
Thonningianin A	*Penthorum chinense*	AD	Aβ- and tau-transgenic C. elegans	AMPK/Nrf2/GPX4 + iron chelation	GPX4 activity/expression ↑, Keap1 degradation, Nrf2 nuclear translocation ↑, iron accumulation ↓; Aβ/tau aggregation ↓	[174] D
			PC12 cells and SH-SY5Y cells; Nrf2-knockout HeLa cells			
Syringin	*Eugenia caryophyllata*	AD	APP/PS1 mice	iASPP-Keap1/Nrf2	Nrf2 stabilization ↑, synaptic plasticity ↑, cognition ↑	[175] I
Water extract of Moschus (WEM)	*Moschus berezovskii*	AD	HT-22 cells (erastin-induced)	Keap1/Nrf2	GPX4 ↑, SLC7A11 ↑, FTH1 ↑, FPN1 ↑, HO-1 ↑; Keap1 ↓, TFRC ↓, MDA ↓, ROS ↓	[176] I
Polycatechols PDP/PLDP	Synthetic polymers (dopamine/L-DOPA backbone)	AD	SH-SY5Y and HEK293T cells	GPX4 restoration, LIP sequestration, Fe^3+^ chelation	Fe^3+^ chelation (PLDP KD ≈ 3 μM), Aβ42/tau aggregation ↓, lipid peroxidation ↓, GSH ↑, MMP ↑	[177] C
Gallic acid-tryptophan cyclodipeptide hybrid (GCTR)	Synthetic molecule (gallic acid backbone)	AD	SH-SY5Y cells	GPX4 activity restoration, Nrf2, Fe chelation	Fe^3+^ chelation (KD = 120 μM), Aβ42/tau aggregation ↓, lipid peroxidation ↓, GPX4 ↑	[178] C

Note: ↑, upregulated or increased; ↓, downregulated or decreased; →, direction of effect. Evidence levels are defined in Section Literature Identification and Scope: D, Direct; S, Supportive; I, Indirect; M, Mixed; C, Complementary or outside the core scope.

**Figure 7:**
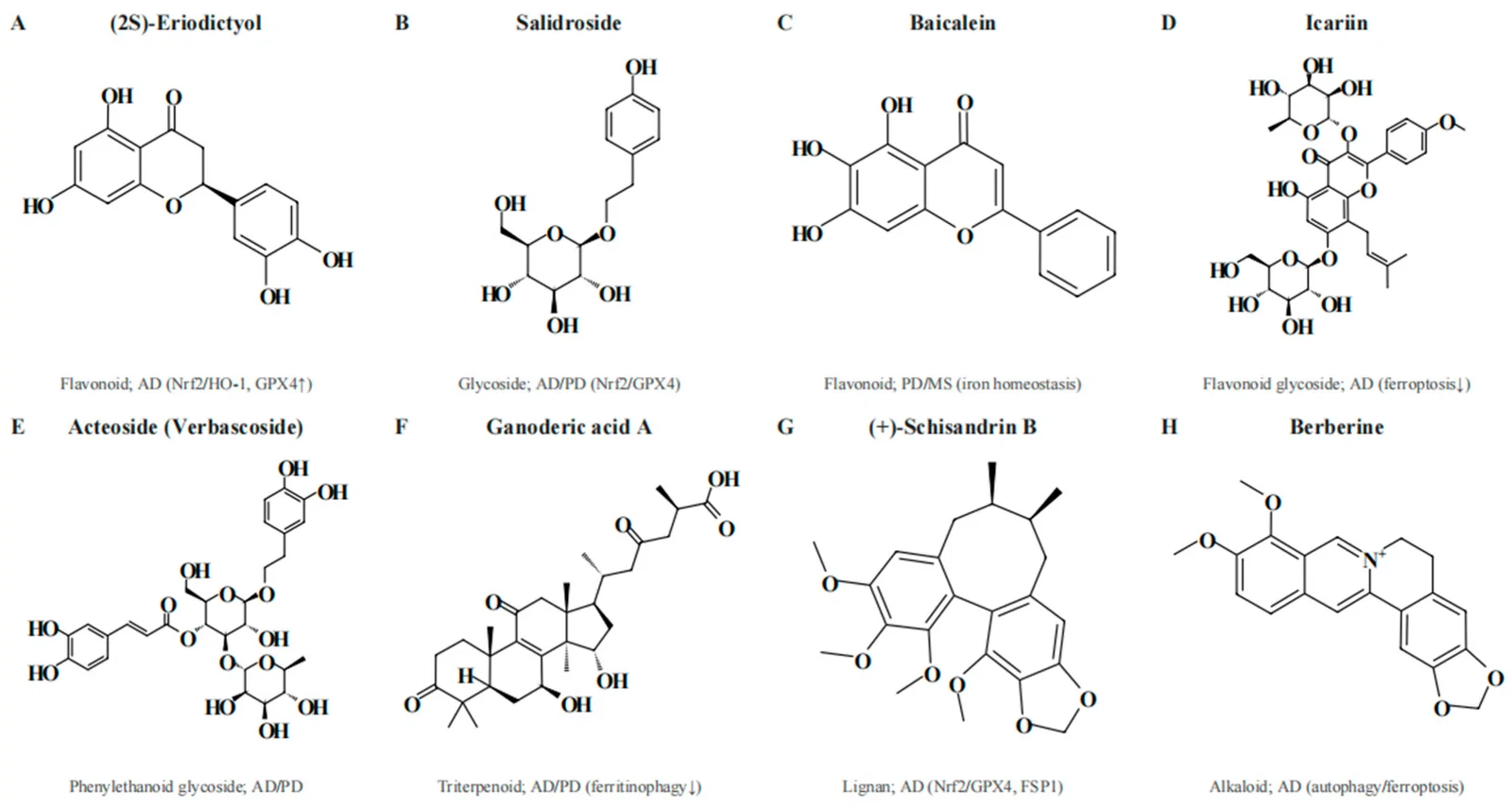
Representative chemical structures of major TCM-derived anti-ferroptotic compounds discussed in Section 4.1: (**A**). eriodictyol (flavonoid; AD); (**B**). salidroside (glycoside; AD/PD); (**C**). baicalein (flavonoid; PD/MS); (**D**). icariin (flavonoid glycoside; AD); (**E**). acteoside (phenylethanoid glycoside; AD/PD); (**F**). ganoderic acid A (triterpenoid; AD/PD); (**G**). schisandrin B (lignan; AD); (**H**). berberine (alkaloid; AD). Structures were obtained from PubChem (https://pubchem.ncbi.nlm.nih.gov/) (accessed on 18 September 2026).

Across the seven structural classes cataloged above, several cross-cutting patterns emerge. First, the Nrf2/GPX4 axis is the most frequently engaged pathway, followed by correction of iron homeostasis through TFR1/FPN1/FTH1/NCOA4 and inhibition of ACSL4-dependent lipid peroxidation, indicating that chemically diverse natural products converge on a limited set of ferroptosis control nodes. Second, the strength of evidence is uneven. Salidroside, ginsenosides, acteoside, and ganoderic acid A are supported by in vivo data in more than one disease model, whereas for most other components the evidence rests on a single cell or animal study. Third, nearly all studies are preclinical, rely on toxin-induced models such as MPTP, 6-OHDA, and Aβ injection, and rarely report dose–response relationships, pharmacokinetics, or brain exposure, which limits cross-study comparison. These patterns suggest that future work should prioritize components with multi-model in vivo evidence and should incorporate pharmacokinetic and dose-finding data at an early stage. The molecular structures of representative compounds with multi-model in vivo evidence are summarized in Figure 7.

Evidence beyond the canonical Nrf2–GPX4 pathway currently comprises FSP1-associated and GPX4-independent observations. Among individual agents, schisandrin B has been reported to cooperate with FSP1-dependent defense [166], whereas wolfberry glycopeptide protected dopaminergic neurons independently of GPX4 without assignment to FSP1, DHODH, or GCH1/BH4 [154]. Direct pathway-specific validation of DHODH/mitochondrial CoQ10, GCH1/BH4, and Nrf2-independent mechanisms in individual TCM-derived compounds remains a priority. ZiYin XiFeng Formula activates Nrf2/SLC7A11/GPX4 without changing Keap1 [179] and is therefore classified as a complex Nrf2-dependent formulation. These findings define specific priorities for screening TCM-derived compounds against FSP1, DHODH, GCH1/BH4, and Nrf2-independent readouts.

### 4.2. TCM Compound Formulas: Multi-Component Network Regulation

TCM compound formulas combine multiple herbs and may influence several ferroptosis-related pathways simultaneously. Formal component-wise comparisons are required to establish effects beyond those of individual herbs. Danggui Shaoyao San inhibits lipid metabolism-associated neuronal ferroptosis through the AMPK/Sp1/ACSL4 axis and improves cognition in APP/PS1 mice [180]. Dingzhen Pills inhibit the cGAS–STING pathway, suppressing neuronal ferroptosis and neuroinflammation in PD mice [181]. ZiYin XiFeng Formula modulates the Nrf2/SLC7A11/GPX4 pathway without a reported change in Keap1 in MPTP mice [179]. SuanZaoRen Decoction activates the DJ-1/Nrf2 pathway and is associated with reduced ferroptosis and neuronal and synaptic injury in AD mice [182]. Linggui Zhugan Decoction reduces AMPK/p53 signaling and restores SLC7A11/GPX4 levels in AD models [183]. Xiao-Xu-Ming Decoction, Tianma Gouteng Decoction, and the YHG mixture (icariin, astragaloside IV, and puerarin) have also been associated with reduced lipid peroxidation or modulation of the System Xc^−^–GPX4–ACSL4–LPCAT3 axis [113,184,185]. These findings support network-level associations (Table 8), while compound-specific attribution requires direct component-wise validation.

**Table 8:** Mechanisms of TCM compound formulas in regulating ferroptosis.

Component	Source	Disease	Model	Signaling Pathway	Key Mechanisms	Ref./ Level
Danggui Shaoyao San	*Angelica sinensis*, *Paeonia lactiflora*, *Ligusticum chuanxiong*, *Atractylodes macrocephala*, *Poria cocos*, *Alisma orientale* (3:16:4:4:8:8, Jin Gui Yao Lue)	AD	APP/PS1 mice	AMPK/Sp1/ACSL4	Activation of p-AMPK → Sp1 dissociates from the ACSL4 promoter → ACSL4 ↓; FTH ↑, GPX4 ↑; brain Fe^2+^/MDA/4-HNE ↓, GSH-Px/T-AOC ↑, improved lipid metabolism	[180] I
Dingzhen Pills	*Astragalus membranaceus*, *Atractylodes macrocephala*, *Paeonia lactiflora*, *Rehmannia glutinosa* (prepared/dried), *Angelica sinensis*, *Ligusticum chuanxiong*, *Gastrodia elata*, *Gentiana macrophylla*, *Clematis chinensis*, *Saposhnikovia divaricata*, *Schizonepeta tenuifolia*, *Asarum heterotropoides*, *Buthus martensii*	PD	MPTP mice	cGAS–STING	cGAS–STING ↓ → ACSL4 ↓, GPX4 ↑, SLC7A11 ↑; midbrain iron/MDA/ROS ↓, TH neurons ↑, α-syn ↓, neuroinflammation ↓	[181] I
			SH-SY5Y cells (MPP^+^)			
ZiYin XiFeng Formula	Prepared *Rehmannia glutinosa*, *Cornus officinalis*, *Gastrodia elata*, *Uncaria rhynchophylla*, *Glycyrrhiza uralensis*, *Paeonia lactiflora*, *Ligusticum chuanxiong*	PD	MPTP mice	Nrf2/SLC7A11/GPX4 (Keap1-independent)	Promotion of Nrf2 nuclear translocation; Nrf2/SLC7A11/GPX4/HO-1 ↑, TFR1 ↓; GSH ↑, MDA ↓, Fe^2+^ ↓, ROS ↓, mitochondrial crista repair	[179] I
			SH-SY5Y cells (MPP^+^)			
SuanZaoRen Decoction	*Ziziphus jujuba*, *Ligusticum chuanxiong*, *Anemarrhena asphodeloides*, *Poria cocos*, honey-fried *Glycyrrhiza uralensis* (Shang Han Lun)	AD	APP/PS1 mice	DJ-1/Nrf2→GPX4/SLC7A11	Activation of DJ-1/Nrf2; GPX4 ↑, SLC7A11 ↑, FPN1 ↑; TFR1 ↓, FTH1 ↓, FTL ↓, ACSL4 ↓, iron deposition ↓, Aβ ↓, synaptic repair	[182] S
Linggui Zhugan Decoction	*Cinnamomum cassia*, *Poria cocos*, *Atractylodes macrocephala*, *Glycyrrhiza uralensis* (3:4:3:2, Zhang Zhongjing)	AD	APP/PS1 mice	AMPK, p53	Reversal of AMPK/p53 elevation; SLC7A11 ↑, GPX4 ↑, NeuN ↑; MDA ↓, Fe^2+^ ↓, GSH ↑, iron deposition ↓	[183] I
Xiao-Xu-Ming Decoction	*Ephedra sinica*, *Ephedra sinica*, *Sinomenium acutum*, etc. (Qian Jin Fang)	AD	HT-22 cells (glutamate-induced)	Inhibition of ferroptosis	Reduced lipid peroxidation, decreased iron aggregation, improved mitochondrial damage	[184] I
Tianma Gouteng Decoction	*Gastrodia elata*, *Uncaria rhynchophylla*, *Haliotis diversicolor*, etc.	PD	PD mice	ACSL4-mediated lipid peroxidation	Inhibition of ACSL4, inhibition of ferroptosis	[185] I
			rotenone-treated PC12 cells			
YHG mixture (icariin + astragaloside IV + puerarin)	*Epimedium brevicornu*, *Astragalus membranaceus*, *Pueraria lobata* (120:80:80 mg/kg)	AD	APP/PS1 mice	System Xc^−^-GPX4-ACSL4-LPCAT3	SLC7A11 ↑, SLC3A2 ↑, GPX4 ↑; ACSL4 ↓, LPCAT3 ↓, brain/serum iron ↓, MDA ↓, Aβ ↓	[113] I

Note: ↑, upregulated or increased; ↓, downregulated or decreased; →, direction of effect. Evidence levels are defined in Section Literature Identification and Scope: D, Direct; S, Supportive; I, Indirect; M, Mixed; C, Complementary or outside the core scope.

### 4.3. Characteristic TCM Therapies: Systemic Regulation Mediated by Physical Stimulation

Beyond pharmacological interventions, characteristic TCM therapies such as acupuncture, moxibustion, and electroacupuncture use physical stimulation of acupoints and have been reported to influence neuroendocrine, immune, and ferroptosis-related signaling in preclinical models. Electroacupuncture at Baihui (GV20) and Yintang (EX-HN3) combined with pricking at Renzhong (GV26) (the Tongdu Qishen acupuncture method) activates neuronal antioxidant function through the p62/Keap1/Nrf2 pathway, upregulates GPX4, reduces PTGS2, and inhibits neuronal ferroptosis in AD mice [186,187]. Electroacupuncture at Baihui (GV20) and Shenting (GV24) reduces Fe^2+^ by upregulating IRP1/FPN1, thereby inhibiting mitochondrial ferroptosis and alleviating cerebral ischemic injury [186,187]. Acupuncture at Baihui (GV20) combined with electroacupuncture at Quchi (LI11) and Zusanli (ST36) protects nigral TH^+^ neurons in PD mice by downregulating DMT1 and upregulating FPN1/GPX4 [186]. The Xingnao Kaiqiao acupuncture method (Neiguan PC6, Renzhong GV26, and Sanyinjiao SP6) has been associated with increased HIF-1α and reduced apoptosis and ferroptosis [187,188]. Scalp point-to-point penetration needling (from Baihui GV20 toward Qubin GB7) upregulates GPX4/FTH1 and reduces iron and MDA through the p62/Keap1/Nrf2 pathway [186,187]. Moxibustion has been reported to improve dopaminergic-neuron survival in the substantia nigra pars compacta of PD rats while reducing ferroptosis-associated changes [189]. These findings support further preclinical evaluation of non-pharmacological TCM interventions.

#### 4.3.1. Electroacupuncture (EA)

Electroacupuncture has been investigated in AD, PD, and stroke models for effects on p62/Keap1/Nrf2, Nrf2–GPX4, and iron-homeostasis pathways (Table 9).

**Table 9:** Acupoints and mechanisms of electroacupuncture in regulating ferroptosis.

Therapy/Acupoints	Disease	Model	Signaling Pathway	Key Evidence	Ref./ Level
EA at Baihui (GV20) and Yintang (EX-HN3) + pricking at Renzhong (GV26) (Tongdu Qishen method)	AD	APPswe/PS1dE9 mice	p62/Keap1/Nrf2	Hippocampal SOD ↑, MDA ↓, Fe ↓, GPX4 mRNA ↑, PTGS2 mRNA ↓; inhibition of ferroptosis	[190,191] I
EA at Baihui (GV20) and Shenting (GV24)	AD; Ischemic stroke	AD mice	Iron metabolism (IRP1/FPN1)	Fe^2+^ ↓, IRP1 ↑, FPN1 ↑; inhibition of mitochondrial ferroptosis; EA preconditioning activates Nrf2 and reduces infarct volume	[101,192] M
EA (Quchi LI11 + Zusanli ST36) + acupuncture at Baihui (GV20)	PD	PD mice	Iron metabolism (DMT1/FPN1) + antioxidant	DMT1 ↓, Bax ↓, FPN1 ↑, GPX4 ↑, Bcl-2 ↑; protection of nigral TH^+^ neurons	[193] I
EA at Baihui (GV20) and Shenshu (BL23) *	AD	D-galactose-induced AD rats	Autophagy (PI3K/Akt/mTOR)	Inhibition of excessive autophagy activation, neuronal protection	[194] C
EA at Baihui (GV20) and Fengfu (GV16) + bilateral Shenshu (BL23) *	AD	AD mice	Anti-apoptosis	Modulation of Bcl-2/Bax/Caspase-3 levels, inhibition of hippocampal neuronal apoptosis	[195] C
EA at Neiguan (PC6), etc.	Ischemic stroke; Myocardial ischemia	—	Nrf2-GPX4	GPX4 ↑, FTH1 ↑; Fe^2+^/ACSL4 ↓, SOD ↑, GSH ↑	[191,196] C
EA at Shenmen (HT7) and Dazhui (GV14)	Hemorrhagic stroke	Rats with cerebral hemorrhage	ROS	Inhibition of ROS accumulation, reduced ferritin and transferrin in hematoma tissue	[102] C
Eye acupuncture	Ischemic stroke	Cerebral ischemia–reperfusion (CIRI) rats	System Xc^−^-GSH-GPX4	Regulation of the System Xc^−^-GSH-GPX4 axis	[103] C

Note: ↑, upregulated or increased; ↓, downregulated or decreased; →, direction of effect. Evidence levels are defined in Section Literature Identification and Scope: D, Direct; S, Supportive; I, Indirect; M, Mixed; C, Complementary or outside the core scope. Entries marked with * primarily report autophagy, apoptosis, pyroptosis, or cuproptosis endpoints.

#### 4.3.2. Acupuncture

Acupuncture has been investigated in models of AD, PD, stroke, and related conditions for effects on ferroptosis-associated autophagy, apoptosis, pyroptosis, and p62/Keap1/Nrf2 signaling (Table 10).

**Table 10:** Acupoints and mechanisms of acupuncture in regulating ferroptosis.

Therapy/Acupoints	Disease	Model	Signaling Pathway	Key Evidence	Ref./ Level
Acupuncture at Baihui (GV20) and Yongquan (KI1) *	AD	Young AD mice	Autophagy	Regulation of autophagy after 21 interventions, neuronal protection	[197] C
Xingnao Kaiqiao acupuncture (Neiguan PC6 + Renzhong GV26 + Sanyinjiao SP6) *	Stroke; AD	—	HIF-1α anti-apoptosis	Upregulation of HIF-1α, anti-apoptosis; combined with alteplase for acute cerebral infarction	[198] C
Scalp point-to-point penetration needling (Baihui GV20 toward Qubin GB7)	Hemorrhagic stroke; AD	Rats with cerebral hemorrhage	p62/Keap1/Nrf2	GPX4 ↑, FTH1 ↑; iron ↓, MDA ↓	[199] C
Tiaoshen acupuncture	AD	APP/PS1 mice	PINK1/Parkin mitophagy	Activation of mitophagy	[200] C
Bushen Huoxue acupuncture *	AD	SAMP8 mice	NLRP1/Caspase-1/IL-1β/IL-18	Inhibition of pyroptosis	[201] C
Scalp point-to-point penetration needling *	AD	AD rats	NF-κB/NLRP3/Caspase-1	Inhibition of pyroptosis	[202] C
“Shugan Tiaoshen” EA (Baihui GV20 + Yintang EX-HN3 + Hegu LI4 + Taichong LR3)	Post-stroke depression	Post-stroke depression rats; rats with comorbid chronic inflammatory pain and depression	Ferroptosis/neuroinflammation	Improvement of depression-like behavior	[203,204] C
“Tongdu Tiaoshen” acupuncture (Dazhui GV14 + Shuigou GV26 + Baihui GV20 + Shenting GV24)	Depression	Depression model rats	Ferroptosis	Regulation of ferroptosis-related pathways	[205] C

Note: ↑, upregulated or increased; ↓, downregulated or decreased; →, direction of effect. Evidence levels are defined in Section Literature Identification and Scope: D, Direct; S, Supportive; I, Indirect; M, Mixed; C, Complementary or outside the core scope. Entries marked with * primarily report autophagy, apoptosis, pyroptosis, or cuproptosis endpoints.

#### 4.3.3. Moxibustion

Moxibustion has been investigated in PD and ischemic-injury models for effects on dopaminergic-neuron survival and ferroptosis-associated markers (Table 11).

**Table 11:** Mechanisms of moxibustion in regulating ferroptosis.

Therapy/Acupoints	Disease	Model	Signaling Pathway	Key Evidence	Ref./ Level
Moxibustion	PD	PD rats	Inhibition of ferroptosis	Increased survival rate of dopaminergic neurons in the SNpc	[189] I
Moxibustion (Baihui and Sishencong)	PD	PD mice	Reduce lipid peroxidation, inhibit ferroptosis	SNX5 ↓, GSH ↑, Fe^2+^ ↓, MDA ↓, GPX4 ↑, FTH1 ↑, GSH/GSSG ratio ↑	[206] I

Note: ↑, upregulated or increased; ↓, downregulated or decreased; →, direction of effect. Evidence levels are defined in Section Literature Identification and Scope: D, Direct; S, Supportive; I, Indirect; M, Mixed; C, Complementary or outside the core scope.

#### 4.3.4. Other Characteristic Therapies

Beyond conventional acupuncture and electroacupuncture, several characteristic or combined approaches have shown anti-ferroptotic or related neuroprotective potential. When applied as manual acupuncture, the Tongdu Qishen method increases GPX4 and decreases PTGS2 in AD rats, consistent with its electroacupuncture effects described above [190,191]. Combining electroacupuncture with knockdown of the long non-coding RNA SNHG1 reduces MDA and iron accumulation and enhances Nrf2 expression in AD mice, suggesting that targeting regulatory RNAs can augment the anti-ferroptotic effect of acupuncture [45]. Eye acupuncture regulates the CTR1/FDX1 pathway and attenuates cuproptosis, another metal-dependent form of regulated cell death, in nerve cells of rats with cerebral ischemia–reperfusion injury [207]. These approaches broaden the interventional scope of characteristic TCM therapies and warrant further mechanistic validation (Table 12).

**Table 12:** Other characteristic therapies in regulating ferroptosis.

Therapy/Acupoints	Disease	Model	Signaling Pathway	Key Evidence	Ref. / Level
EA with lncRNA SNHG1 knockdown	AD	AD mice	Nrf2	Reduced MDA/iron, increased GSH, enhanced Nrf2 expression	[45] I
Eye acupuncture *	Cerebral ischemia–reperfusion injury	CIRI rats	CTR1/FDX1 cuproptosis	Regulation of cuproptosis-related pathways, neuroprotection	[207] C

Note: ↑, upregulated or increased; ↓, downregulated or decreased; →, direction of effect. Evidence levels are defined in Section Literature Identification and Scope: D, Direct; S, Supportive; I, Indirect; M, Mixed; C, Complementary or outside the core scope. Entries marked with * primarily report autophagy, apoptosis, pyroptosis, or cuproptosis endpoints.

## 5. Animal-Model Evidence Base, Limitations, and Future Directions

Animal models have served a dual role in the work summarized here. On the one hand, they provided key experimental evidence that ferroptosis contributes to neurodegeneration; on the other, they constitute the experimental platform on which almost all anti-ferroptotic TCM interventions catalogued in this review have been evaluated. This section consolidates the animal-model evidence base, delineates the current limitations of model-based research, and outlines model-centered priorities for translation toward clinical application.

### 5.1. Animal Models Provide a Conserved Ferroptotic Phenotype and an In Vivo Testing Platform for TCM

Across the five diseases considered in this review, model families that differ widely in induction strategy, including genetic, toxin-based, protein-injection, and inflammatory approaches, all reproduce the ferroptotic signature documented in patients, namely iron accumulation, GSH/GPX4 depletion, ACSL4 elevation, and accumulation of lipid peroxidation products such as 4-HNE and MDA [48,53,54,58,59,67,68]. In AD, APP/PS1, 5xFAD, 3×Tg, and SAMP8 mice, as well as Aβ-injection and ICV-STZ models, recapitulate hippocampal iron overload and memory-relevant ferroptotic changes. In particular, conditional ablation of Gpx4 in forebrain neurons is sufficient to reproduce memory impairment, thereby providing genetic evidence that loss of ferroptosis defense drives the phenotype [59,106,110,112]. In PD, MPTP/MPP+, 6-OHDA, rotenone, and α-synuclein-driven models (SNCA A53T and AAV-α-synuclein) reproduce nigral iron accumulation and GPX4/GSH impairment [48,67,68]. In ALS, GPX4 deficiency is a shared early feature of SOD1G93A, TDP-43, and C9orf72 models; restoring GPX4 delays onset and prolongs survival in SOD1G93A mice, whereas neuron-specific Gpx4 ablation causes paralysis [86,87,88]. In MS, cuprizone-induced oligodendrocyte ferroptosis and the GPX4-low/ACSL4-high signature of EAE position ferroptosis upstream of demyelination and neurodegeneration [95,96,97]. In HD, striatal and cortical iron accumulation in R6/2 mice, together with the rescue of 3-nitropropionic acid-induced striatal death by ferroptosis inhibitors, links iron dyshomeostasis to the ferroptotic execution machinery [73,74,78,79].

Three properties of these systems make them indispensable for TCM research. First, they enable causal pharmacology. Genetic manipulation of GPX4 or ACSL4, as well as treatment with selective ferroptosis inhibitors, shifts both biochemical markers and pathological outcome, thereby establishing ferroptosis as an intervention point rather than an epiphenomenon [59,87,88,97].

Second, they provide functional endpoints of clinical significance that cell systems cannot offer, including spatial memory in AD models, motor performance in PD models, survival in ALS mice, and myelination status in MS models. These outcomes are precisely the readouts through which TCM interventions have been judged beneficial. Third, they integrate whole-organism processes relevant to the multi-target character of TCM, such as blood–brain barrier penetration, systemic iron handling, and neuroimmune crosstalk.

The weight of this in vivo evidence is substantial. Across the twelve evidence tables of this review (Table 1, Table 2, Table 3, Table 4, Table 5, Table 6, Table 7, Table 8, Table 9, Table 10, Table 11 and Table 12), approximately 55% of the experimental entries involve in vivo models, spanning more than 30 distinct model platforms, whereas the remainder represent in vitro mechanistic complements.

Representative bioactive components, including eriodictyol, icariin, salidroside, acteoside, and ganoderic acid A, have been validated in multiple independent in vivo models, and some have been tested across more than one disease, for instance in APP/PS1 and SAMP8 mice for AD and in MPTP, 6-OHDA, and rotenone models for PD [106,112,130,131,132,133,143,144,157,158]. Compound formulas (Danggui Shaoyao San, ZiYin XiFeng formula, and SuanZaoRen decoction) and characteristic therapies (electroacupuncture, acupuncture, and moxibustion) likewise improve functional deficits together with ferroptotic markers in APP/PS1, MPTP, and cuprizone-based systems [179,180,181,182,183,189,190,191]. Invertebrate in vivo models, such as Drosophila PD models, add fast complementary screening capacity within the same framework [125]. The consistency with which pharmacologically and genetically distinct TCM interventions restore GPX4/GSH, correct iron flux, and reduce lipid peroxidation, while also improving functional outcomes across model families, provides convergent, though predominantly preclinical, support for a shared anti-ferroptotic mechanism.

### 5.2. Current Limitations of the Model-Based Evidence

Despite this breadth, several limitations constrain the interpretation and translation of the animal-model evidence. First, model fidelity remains incomplete. Toxin- and injection-based models (MPTP, 6-OHDA, Aβ, and ICV-STZ) capture selective ferroptotic phenotypes but only partially reproduce the chronic, proteinopathy-driven pathology of human disease, and even genetic models do not fully mirror human aging-related neurodegeneration [67,86]. Second, attribution of efficacy to ferroptosis inhibition is often indirect. Ferroptosis coexists and crosstalks with apoptosis, pyroptosis, necroptosis, and cuproptosis, and several studies have not used selective ferroptosis inhibitors or ferroptosis-specific genetic controls to demonstrate that the observed protection is ferroptosis-dependent [12,13,14]. Third, reliable in vivo ferroptosis biomarkers remain scarce. Quantitative detection of lipid peroxidation products or GPX4 activity in intact tissue is technically demanding, which hampers the longitudinal assessment of drug effects and translation to patient stratification [208].

A row-level classification of the 116 experimental entries yielded 13 Direct, 10 Supportive, 62 Indirect, 11 Mixed, and 20 Complementary classifications. Direct examples include loss- or gain-of-function tests of VDR/Nrf2, NOX4, NCOA4, IRP2, GPX4, GSK3β, or AMPK/Nrf2 together with lipid-peroxidation readouts. By contrast, studies in which ferrostatin-1, liproxstatin-1, or an inducer only benchmarked the disease model, but did not reverse or abolish the tested TCM intervention, were graded as Supportive rather than Direct. Rows that combine evidence from different levels or scopes are graded as Mixed. This approach prevents changes in ROS, MDA, GSH, GPX4, SLC7A11, or Nrf2 alone from being interpreted as proof of ferroptosis-dependent efficacy.

Fourth, on the TCM side, most studies still employ single doses and short treatment windows and rarely report dose–response relationships, pharmacokinetics, or brain exposure. Inconsistencies in dosage, treatment duration, dosage form, and quality control across studies further weaken cross-study comparability. Characteristic therapies face additional methodological constraints, including non-uniform acupoint specificity and stimulation parameters, as well as the inherent difficulty of designing sham controls within blinding frameworks. Finally, coverage across diseases is uneven. Although AD and PD models dominate the in vivo TCM literature, comparatively few interventions have been evaluated in genetic or symptomatic models of HD, ALS, and MS, which limits the generalization of this framework. In addition, several translational caveats deserve emphasis: publication bias toward positive results cannot be excluded in this literature; blood–brain barrier penetration and pharmacokinetic exposure are rarely verified for TCM components; formulation variability, potential toxicity (for example, the narrow therapeutic window of celastrol), and herb–drug interactions remain insufficiently characterized; and no validated human ferroptosis biomarker is currently available for patient stratification or treatment monitoring.

Beyond these experimental issues, the standardization of TCM materials itself constitutes a central translational barrier. For herbal preparations, reproducibility depends on botanical authentication (verification of species and plant part by macroscopic, microscopic, and DNA-barcoding methods), geographical origin (including daodi producing regions), harvesting season, processing method (paozhi), extraction solvent and procedure, and batch-to-batch quality control through chemical fingerprinting (for example, HPLC or UPLC-MS/MS fingerprints) together with quantification of marker compounds. Most studies cataloged in this review report only minimal information on these parameters, which limits reproducibility and cross-study comparison. Future studies should follow established reporting guidelines for herbal research and should deposit chemical fingerprints and marker-compound content for each batch used.

### 5.3. Model-Centered Directions for Future Research

Priorities for strengthening the animal-model evidence base should therefore include the following measures. First, orthogonal validation. Interventions should be tested in complementary genetic and toxin-based models of the same disease, with selective ferroptosis inhibitors (for example, ferrostatin-1 and liproxstatin-1) or pathway-specific genetic manipulation serving as positive controls, so that neuroprotection can be causally attributed to ferroptosis inhibition. Second, standardized multi-model readouts. Biochemical ferroptosis markers should be combined with functional endpoints and, where possible, with in vivo iron imaging to permit quantitative and longitudinal assessment. Third, expanded disease coverage. Genetic and clinically relevant models of HD, ALS, and MS, including proteinopathy and inflammatory variants, should receive greater attention in future TCM studies. Fourth, drug-development-oriented designs. Dose–response evaluation, pharmacokinetic profiling, brain-exposure measurement, and safety windows (for example, for components such as celastrol with narrow therapeutic margins) should be incorporated into animal studies at an early stage. Fifth, mechanistic resolution. The contributions of GPX4-independent defense axes, including the FSP1/CoQ10 and DHODH pathways, and the temporal relationship between ferroptotic features and disease progression should be dissected in disease-relevant models [12,13,14,208].

Finally, clinical translation must be informed by completed and ongoing trials of ferroptosis-targeting strategies in neurodegenerative diseases. The brain-permeable iron chelator deferiprone was tested in early Parkinson’s disease in the phase II FAIRPARK-II trial (NCT02655315), which reduced nigrostriatal iron but unexpectedly worsened motor outcomes over 36 weeks [209]. An earlier pilot trial (NCT00943748) reported motor improvement under a conservative chelation regimen, although its small sample and delayed-start design limit inference [210]. In Alzheimer’s disease, the phase II 3D trial (NCT03234686) reduced hippocampal iron but accelerated cognitive decline [211]. In pantothenate kinase-associated neurodegeneration, deferiprone reduced brain iron and showed a trend toward slower progression in the TIRCON2012V1 trial (NCT01741532) [212]. Together, these results show that lowering brain iron alone is not a validated surrogate for clinical benefit and may be harmful in some settings. Future trials therefore require ferroptosis-specific biomarkers, careful patient stratification, and interventions that address downstream phospholipid peroxidation without indiscriminate systemic iron removal.

Together, these model-centered refinements, coupled with chemical benchmarking, standardized formulation control, and clinically validated ferroptosis biomarkers, will provide the robust preclinical foundation required to translate the anti-ferroptotic potential of TCM into clinically meaningful therapies for neurodegenerative diseases.

## 6. Conclusions

Ferroptosis is an iron-dependent form of regulated cell death executed when iron-enabled phospholipid peroxidation overwhelms GPX4-dependent and parallel antioxidant defenses. Disrupted iron handling, accumulation of oxidized phospholipids, and inadequate ferroptosis-defense capacity recur across Alzheimer’s disease, Parkinson’s disease, Huntington’s disease, amyotrophic lateral sclerosis, and multiple sclerosis. Mitochondrial metabolism can amplify or restrain these processes in a stimulus- and cell-type-dependent manner. The available data therefore support ferroptosis as a convergent and potentially druggable mechanism, while causal attribution in individual disease models still requires selective rescue or genetic validation.

Against this mechanistic background, TCM represents a source of candidate interventions. Bioactive components of individual herbs, multi-component formulas, and non-pharmacological therapies such as electroacupuncture, acupuncture, and moxibustion have been reported to modulate ferroptosis-associated pathways in preclinical models. The recurring targets include the Nrf2/GPX4 antioxidant axis, TFR1/FPN1/FTH1/NCOA4-mediated iron handling, ACSL4/LPCAT3-mediated lipid peroxidation, and transcriptional regulators such as Nrf2 and p53. These patterns support a network-level mechanistic framework that now requires compound-specific causal validation and clinical confirmation.

Clinical translation will depend on strengthened clinical validation, defined bioavailability and brain exposure, improved methodological consistency, standardized formulation quality, and validated human ferroptosis biomarkers. Future work should combine chemical benchmarking and standardized production with dose–exposure studies, pathway-specific causal tests, biomarker development, and rigorously designed clinical trials. Such work is needed to determine which anti-ferroptotic TCM strategies are reproducible, mechanistically specific, safe, and clinically useful.

## Data Availability

No new data were created or analyzed in this study. Data sharing is not applicable to this article.

## Abbreviations

The following abbreviations are used in this manuscript:

4-HNE	4-hydroxynonenal
6-OHDA	6-hydroxydopamine
Aβ	amyloid-β
ACO1	aconitase 1
ACSL4	acyl-CoA synthetase long-chain family member 4
AD	Alzheimer’s disease
AIFM2	apoptosis-inducing factor mitochondria-associated 2
ALS	amyotrophic lateral sclerosis
AMPK	AMP-activated protein kinase
APP	amyloid precursor protein
ARE	antioxidant response element
BACE1	beta-site amyloid precursor protein cleaving enzyme 1
BDNF	brain-derived neurotrophic factor
BH4	tetrahydrobiopterin
CAG	cytosine-adenine-guanine
CAT	catalase
CD98	cluster of differentiation 98
cGAS	cyclic GMP-AMP synthase
CISD1	CDGSH iron–sulfur domain protein 1
CNKI	China National Knowledge Infrastructure
CoQ10	coenzyme Q10
CR3	complement receptor 3
CTR1	copper transporter 1
DAT	dopamine transporter
DFO	deferoxamine
DHODH	dihydroorotate dehydrogenase
DJ-1	protein/nucleic acid deglycase DJ-1 (PARK7)
DMT1	divalent metal transporter 1
EA	electroacupuncture
EAE	experimental autoimmune encephalomyelitis
EGCG	epigallocatechin gallate
ERK	extracellular signal-regulated kinase
FDX1	ferredoxin 1
FPN (FPN1)	ferroportin 1
FSP1	ferroptosis suppressor protein 1
FTH (FTH1)	ferritin heavy chain 1
FTL	ferritin light chain
FtMt	mitochondrial ferritin
GBD	Global Burden of Disease
GCH1	GTP cyclohydrolase 1
GCLC/GCLM	glutamate-cysteine ligase catalytic/modifier subunit
GCTR	gallic acid-tryptophan cyclodipeptide hybrid
GPX1	glutathione peroxidase 1
GPX4	glutathione peroxidase 4
GSH	glutathione
GSH-Px	glutathione peroxidase
GSK-3β	glycogen synthase kinase 3 beta
GSSG	oxidized glutathione
HD	Huntington’s disease
HIF-1α	hypoxia-inducible factor 1 alpha
HO-1	heme oxygenase-1
HTT	huntingtin
iASPP	inhibitor of apoptosis-stimulating protein of p53
IC50	half-maximal inhibitory concentration
ICS II	icariside II
ICV	intracerebroventricular
IKK	IκB kinase
IL-1β/IL-18	interleukin-1 beta/interleukin-18
IREB2	iron-responsive element-binding protein 2
IRP1/IRP2	iron regulatory protein 1/2
JC-1	mitochondrial membrane potential-sensitive fluorescent probe
JNK	c-Jun N-terminal kinase
KD	dissociation constant
Keap1	Kelch-like ECH-associated protein 1
LC3B	microtubule-associated protein 1 light chain 3 beta
LIP	labile iron pool
lncRNA	long non-coding RNA
LOX	lipoxygenase
LPCAT3	lysophosphatidylcholine acyltransferase 3
LPS	lipopolysaccharide
MAPK	mitogen-activated protein kinase
MCAO	middle cerebral artery occlusion
MDA	malondialdehyde
MDM2	murine double minute 2
MMP	mitochondrial membrane potential
MPP+	1-methyl-4-phenylpyridinium
MPTP	1-methyl-4-phenyl-1,2,3,6-tetrahydropyridine
MRI	magnetic resonance imaging
MS	multiple sclerosis
mTOR	mechanistic target of rapamycin
NCOA4	nuclear receptor coactivator 4
NDs	neurodegenerative diseases
NeuN	neuronal nuclear protein
NF-κB	nuclear factor kappa B
NFTs	neurofibrillary tangles
NLRP1/NLRP3	NOD-like receptor family pyrin domain-containing protein 1/3
NOX	NADPH oxidase
NQO1	NAD(P)H quinone oxidoreductase 1
Nrf2	nuclear factor erythroid 2-related factor 2
OABL	1,6-O,O-diacetylbritannilactone
OGD/R	oxygen–glucose deprivation/reoxygenation
p53	tumor protein p53
p62	sequestosome 1
PD	Parkinson’s disease
PEBP1	phosphatidylethanolamine-binding protein 1
PI3K	phosphoinositide 3-kinase
PINK1	PTEN-induced kinase 1
PL-OH	phospholipid alcohol
PL-OOH	phospholipid hydroperoxide
PSD95	postsynaptic density protein 95
PTGS2	prostaglandin-endoperoxide synthase 2
PUFA	polyunsaturated fatty acid
PUFA-PL	polyunsaturated fatty acid-containing phospholipid
ROS	reactive oxygen species
RSL3	RAS-selective lethal compound 3 (GPX4 inhibitor)
SAMP8	senescence-accelerated mouse prone 8
SIRT1/SIRT3	sirtuin 1/sirtuin 3
SLC3A2	solute carrier family 3 member 2
SLC7A11 (xCT)	solute carrier family 7 member 11
SNHG1	small nucleolar RNA host gene 1
SNpc	substantia nigra pars compacta
SOD	superoxide dismutase
SPP1	secreted phosphoprotein 1
Sp1	specificity protein 1
Src	proto-oncogene tyrosine-protein kinase Src
STEAP3	six-transmembrane epithelial antigen of prostate 3
STING	stimulator of interferon genes
STZ	streptozotocin
T-AOC	total antioxidant capacity
TCM	traditional Chinese medicine
TCR	T-cell receptor
TDP-43	TAR DNA-binding protein 43
TF (Tf)	transferrin
TFA	total flavonoids of Astragalus
TFR1 (TFRC, TfR1)	transferrin receptor 1
TH	tyrosine hydroxylase
TLR4	toll-like receptor 4
TNF-α	tumor necrosis factor alpha
TRAF6	TNF receptor-associated factor 6
TSG	2,3,5,4′-tetrahydroxystilbene-2-O-beta-D-glucoside
UQCRC1	ubiquinol-cytochrome c reductase core protein 1
VDAC	voltage-dependent anion channel
VDR	vitamin D receptor
WEM	water extract of Moschus
WHO	World Health Organization
YHG	icariin-astragaloside IV-puerarin mixture
α-syn	alpha-synuclein
